# Trends in Molecular Diagnostics and Genotyping Tools Applied for Emerging *Sporothrix* Species

**DOI:** 10.3390/jof8080809

**Published:** 2022-07-31

**Authors:** Jamile Ambrósio de Carvalho, Ruan Campos Monteiro, Ferry Hagen, Zoilo Pires de Camargo, Anderson Messias Rodrigues

**Affiliations:** 1 Laboratory of Emerging Fungal Pathogens, Department of Microbiology, Immunology, and Parasitology, Discipline of Cellular Biology, Federal University of São Paulo (UNIFESP), São Paulo 04023062, Brazil; jamileambrosio@hotmail.com (J.A.d.C.); ruanmonteirolj@gmail.com (R.C.M.); zpcamargo1@gmail.com (Z.P.d.C.); 2 Department of Medical Mycology, Westerdijk Fungal Biodiversity Institute, Uppsalalaan 8, 3584 CT Utrecht, The Netherlands; f.hagen@gmail.com; 3 Institute for Biodiversity and Ecosystem Dynamics (IBED), University of Amsterdam, Sciencepark 904, 1098 XH Amsterdam, The Netherlands; 4 Department of Medical Microbiology, University Medical Center Utrecht, Heidelberglaan 100, 3584 CX Utrecht, The Netherlands; 5 Department of Medicine, Discipline of Infectious Diseases, Federal University of São Paulo (UNIFESP), São Paulo 04023062, Brazil

**Keywords:** sporotrichosis, *Sporothrix*, molecular diagnostics, molecular epidemiology, diagnosis, zoonosis, emerging mycosis

## Abstract

Sporotrichosis is the most important subcutaneous mycosis that affects humans and animals worldwide. The mycosis is caused after a traumatic inoculation of fungal propagules into the host and may follow an animal or environmental transmission route. The main culprits of sporotrichosis are thermodimorphic *Sporothrix* species embedded in a clinical clade, including *S.* *brasiliensis*, *S.* *schenckii*, *S.* *globosa*, and *S. luriei*. Although sporotrichosis occurs worldwide, the etiological agents are not evenly distributed, as exemplified by ongoing outbreaks in Brazil and China, caused by *S.* *brasiliensis* and *S.* *globosa*, respectively. The gold standard for diagnosing sporotrichosis has been the isolation of the fungus in vitro. However, with the advance in molecular techniques, molecular assays have complemented and gradually replaced the classical mycological tests to quickly and accurately detect and/or differentiate molecular siblings in *Sporothrix*. Nearly all techniques available for molecular diagnosis of sporotrichosis involve PCR amplification, which is currently moving towards detecting *Sporothrix* DNA directly from clinical samples in multiplex qPCR assays. From an epidemiological perspective, genotyping is key to tracing back sources of *Sporothrix* infections, detecting diversity in outbreak areas, and thus uncovering finer-scale epidemiological patterns. Over the past decades, molecular epidemiological studies have provided essential information to policymakers regarding outbreak management. From high-to-low throughput genotyping methods, MLSA, AFLP, SSR, RAPD, PCR-RFLP, and WGS are available to assess the transmission dynamics and sporotrichosis expansion. This review discusses the trends in the molecular diagnosis of sporotrichosis, genotyping techniques applied in molecular epidemiological studies, and perspectives for the near future.

## 1. A Primer on *Sporothrix* and Sporotrichosis

**Figure 1:**
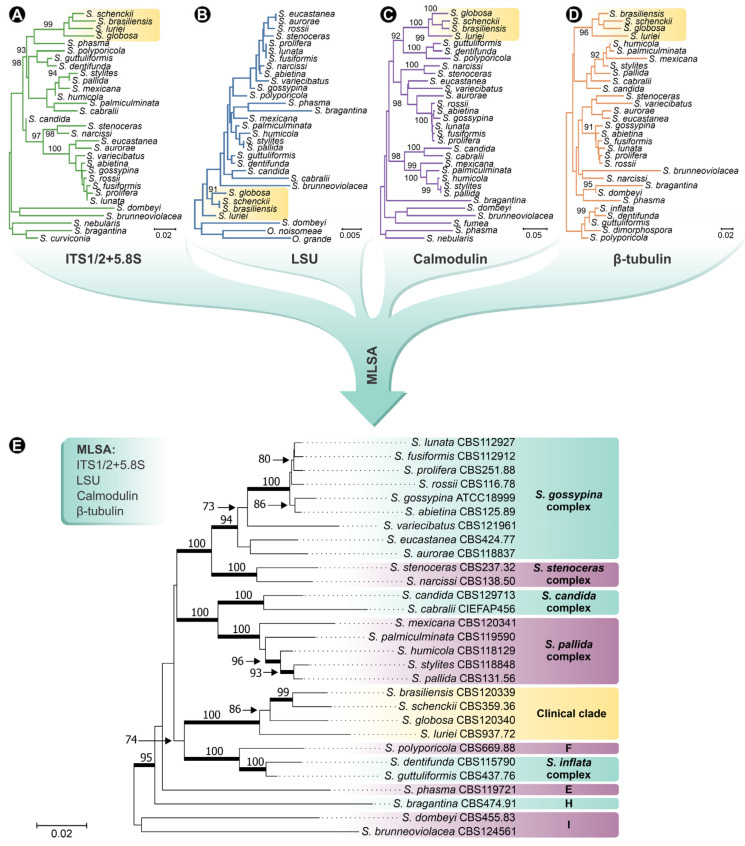
Phylogenetic analysis of *Sporothrix* species. Phylogenetic tree inferred using the Neighbor-Joining method, with 1000 bootstrap replicates performed in MEGA 7, based on (**A**) the internal transcribed spacer (ITS) sequences; (**B**) partial sequences of the large subunit (LSU) of the rRNA; (**C**) partial sequences of the calmodulin (CAL) gene; and (**D**) partial sequences of the β-tubulin (BT2) gene of *Sporothrix* isolates. (**E**) Phylogeny reconstruction based on concatenated sequences of ITS, LSU, CAL, and BT2. Numbers close to the branches represent bootstraps values. Sequences were collected from GenBank (Supplementary Table S1).

Sporotrichosis is a cutaneous or subcutaneous mycosis of mammals caused by *Sporothrix* species, usually affecting the skin and mucous membranes [1]. Benjamin R. Schenck reported the disease for the first time in 1898 [2]. Two years after the isolation of the fungus, in 1900, Hektoen proposed the genus *Sporothrix* [3]. For over a century, the classical agent *S. schenckii* was considered the unique culprit of sporotrichosis, but in 2007, Marimon et al. [4,5], through phylogenetic analysis of calmodulin (exons 3–5), proposed to split *S. schenckii* into several molecular siblings. Currently, *Sporothrix* comprises 53 species divided into clinical and environmental clades (Figure 1). So far, the clinical clade comprises four species causing human and animal infections: *S. brasiliensis*, *S. schenckii* *sensu stricto*, *S. globosa*, and *S. luriei*. Most *Sporothrix* species are embedded in the environmental clade (i.e., *S. pallida*, *S. stenoceras*, *S. inflata*, *S. humicola*, etc.) and present lower pathogenic potential toward mammals. Environmental *Sporothrix* species are usually associated with decaying wood, plant debris, soil, insects, etc. [1,6,7,8,9]. Therefore, the drivers of the emergence of pathogenicity in a genus whose core is mostly saprophytic is an intriguing phenomenon which is still poorly understood [10,11,12,13,14,15,16].

*Sporothrix* species undergo a thermodimorphic transition, facilitating the establishment of infection. The pathogen can be found in the environment at room temperature (25–28 °C) in the filamentous form and as a yeast when infecting the warm-blooded host (36–37 °C) [17,18,19]. The infection can occur through two main routes: the classical or sapronotic and the alternative or animal route. Both are associated with the traumatic inoculation of *Sporothrix* propagules into cutaneous and subcutaneous tissue. During sapronosis, contaminated plants are well-recognized transmission sources, while scratches and/or bites from infected animals play a major role in transmitting the disease to other animals and humans [18,20,21].

**Figure 2:**
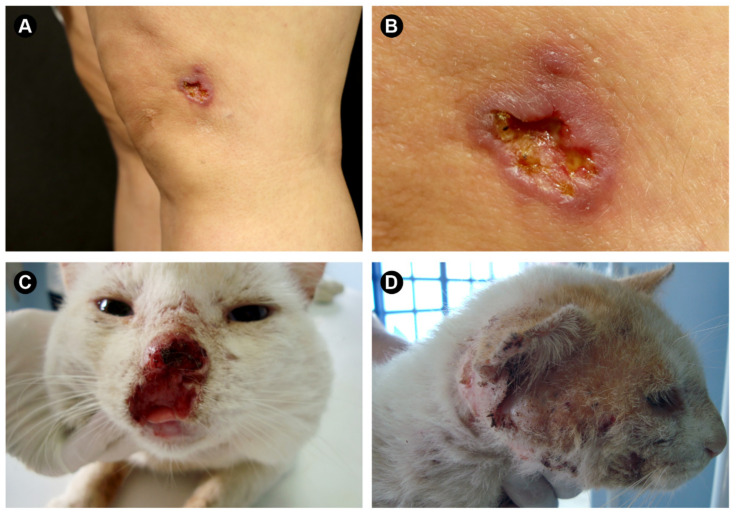
Human and feline sporotrichosis. (**A**,**B**) Human sporotrichosis on the left knee. (**C**,**D**) Feline sporotrichosis with lesions in the nasal and cephalic region. Images of cats with sporotrichosis were kindly provided by Prof. Dr. Mario Augusto Ono (State University of Londrina, Brazil).

In humans, the lesions of the cutaneous form of the disease develop at sites of skin injury and appear as an erythematous, ulcerated, or verrucous nodule (Figure 2A,B). Subsequent nodular lymphangitic spread is a common development (75–90%) [22]. Pulmonary sporotrichosis presumably results from inhalation of the fungus and has been rarely reported [23,24]. The infection may also be hematogenously disseminated and involves the bones, joints, skin, eyes, central nervous system, and/or genitourinary tract [25,26]. However, pulmonary sporotrichosis and hematogenous dissemination are rarely seen in the immunocompetent host but are usually linked to immunosuppression [26,27,28,29].

Animal sporotrichosis has been reported in diverse warm-blooded hosts such as armadillos, camels, cats, cows, dogs, dolphins, mice, etc. [20,30,31,32,33]. However, the domestic cat is the animal most susceptible to infection, and its entry into the sporotrichosis transmission chain represented a meaningful change in the epidemiological picture [34]. The manifestations in cats range from a single skin lesion to fatal disseminated systemic forms. Generally, multiple ulcerative lesions are observed in the cephalic zone, mainly in the nose and paw region (Figure 2C,D) [35,36,37].

Sporotrichosis has a worldwide distribution, although distinct etiological agents are not evenly distributed [5,38,39]. *Sporothrix brasiliensis* occurs in a restricted area of South America, with most cases originating from Brazil since the onset of cat-transmitted outbreaks [1,40,41,42]. Notwithstanding, recent reports demonstrate the presence of this highly virulent species in neighboring countries such as Argentina and Paraguay [43,44] and suspected cases occurring in Bolivia, Colombia, and Panama [45,46]. A human case was recently reported in the UK associated with the importation of pets and global travel [47]. *Sporothrix schenckii*, on the other hand, follows a sapronotic route and is widely distributed across Africa, the Americas, and Oceania [48,49]. Likewise, *S. globosa* shows a global distribution, but large sapronosis occurs in Asia, mainly in India and China, with a high prevalence in Jilin province [18,50]. Species embedded in the *S. pallida* and *S. stenoceras* complexes have sparsely been reported from clinical cases worldwide [6,11,12,13,51].

Judging from the pieces of information above, it is evident that taxonomic developments have promoted important advances in epidemiological scenarios, parasite–host interactions, sensitivity to antifungal agents, and the biology of these pathogens. Therefore, it is imperative to recognize *Sporothrix* species in the clinical scenario [52,53,54,55,56,57,58,59,60,61,62,63]. The diagnosis of sporotrichosis down to genus or species level can bring benefits to treatment and thus impact the clinical outcome of patients, and in the case of the felines, avoid the dispersion of the fungus into the feline and human populations [32,64].

## 2. Laboratorial Diagnostics of Sporotrichosis

**Figure 3:**
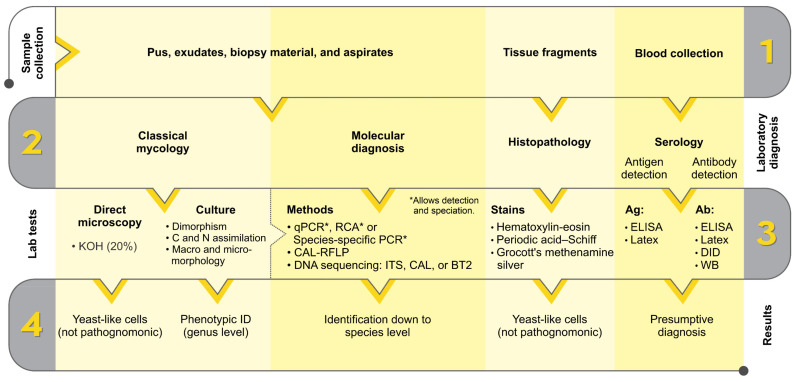
Flowchart for laboratory diagnosis of sporotrichosis. KOH: potassium hydroxide; C: carbon source; N: nitrogen source; qPCR: quantitative polymerase chain reaction; RCA: rolling circle amplification; PCR-RFLP: polymerase chain reaction-restriction fragment length polymorphism; ITS: internal transcribed spacer; CAL: calmodulin; BT2: β-tubulin; Ag: antigen detection; Ab: antibody detection; DID: double immunodiffusion; WB: Western blot.

The diagnosis of sporotrichosis combines clinical, epidemiological, and laboratory data, including direct examination, culture, histopathological and serological tests [65,66] (Figure 3).

A direct mycological examination using potassium hydroxide (KOH), or differential staining, was used to observe the yeast-like cells directly from specimens collected from humans or animals. In the cutaneous form of human sporotrichosis, there are so few organisms present in pus, exudates, biopsy material, and aspirates that, in general, direct examination of such material is unrewarding [65,67,68] unless using immunofluorescence methods [69]. On the other hand, the high fungal load present in the lesions of cats facilitates the direct visualization of budding yeast cells, generally round and oval, often elongated (cigar-shaped cells) [32,37,70,71].

**Figure 4:**
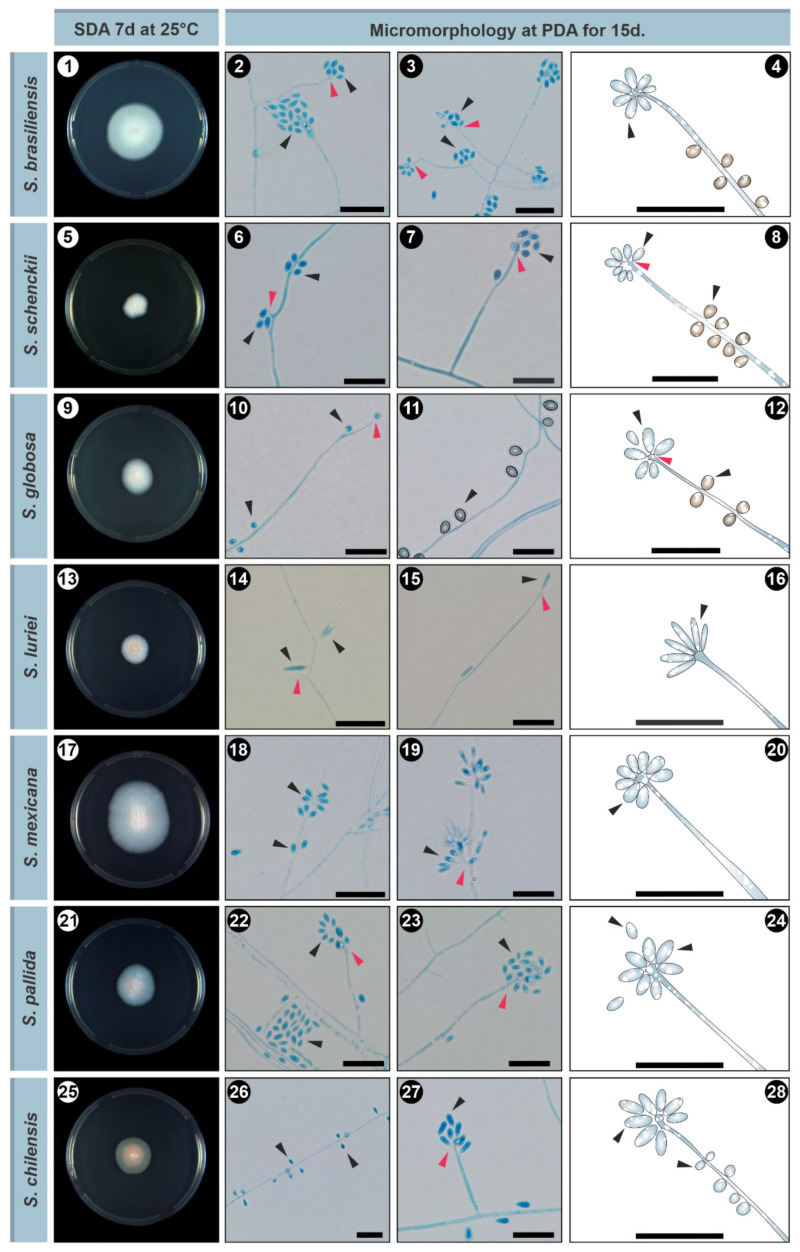
Macromorphological and micromorphological aspects of *Sporothrix* species. (1) Macro- and (2–4) micromorphology of *S. brasiliensis*; (5) Macro- and (6–8) micromorphology of *S. schenckii*; (9) Macro- and (10–12) micromorphology of *S. globosa*; (13) Macro- and (14–16) micromorphology of *S. luriei*; (17) Macro- and (18–20) micromorphology of *S. mexicana*; (21) Macro- and (22–24) micromorphology of *S. chilensis*; (25) Macro- and (26–28) micromorphology of *S. pallida*. Red arrows indicate phialides and black arrows indicate conidia. SDA: Sabouraud dextrose agar. Bar = 10 µm.

**Figure 5:**
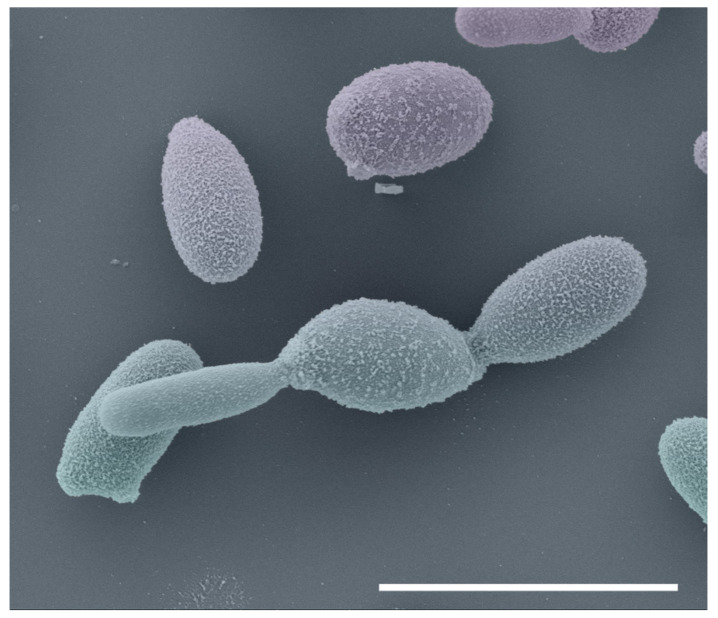
Scanning electron microscopy of *Sporothrix brasiliensis* yeasts cells (BHI broth, seven days, 37 °C, 100 rpm). Bar = 5 µm.

The reference method for diagnosing sporotrichosis is in vitro cultivation of clinical specimens, and these samples are usually taken from lesions, pus, secretions, or biopsies [14]. Samples are normally seeded onto Sabouraud dextrose agar (SDA) and mycosel agar for 7–21 days at room temperature. Macroscopic examination of cultures at room temperature can initially note small and creamy cultures that may turn brown or almost black (Figure 4). With microscopic investigation, it is possible to observe thin, septate hyaline hyphae (1–2 μm wide) with single-celled primary conidia (2.5–5.5 × 1.5–2.5 μm) grouped sympodially in a daisy-like arrangement [12,72] (Figure 4). A second conidal form produced by some strains consists of sessile tick-walled hyaline or brown conidia (2.5–5.5 × 1.5–2.5 μm) that emerge alongside the undifferentiated hyphae. Due to the thermodimorphic nature of *Sporothrix* species, it is recommended to cultivate the fungus on brain–heart infusion (BHI) agar and incubate it at 35–37 °C to develop yeast cells [18,65]. At elevated temperatures, the colonies are initially creamy to gray–yellow after five days of incubation. Microscopically, it comprises round or oval cigar-shaped cells (2–4 × 6 μm), typically bearing terminal blastoconidia (Figure 5) [12,20,73]. There is a significant morphological overlap in *Sporothrix*, thus, speciation based solely on these phenotypic traits is not recommended [13,74,75].

**Figure 6:**
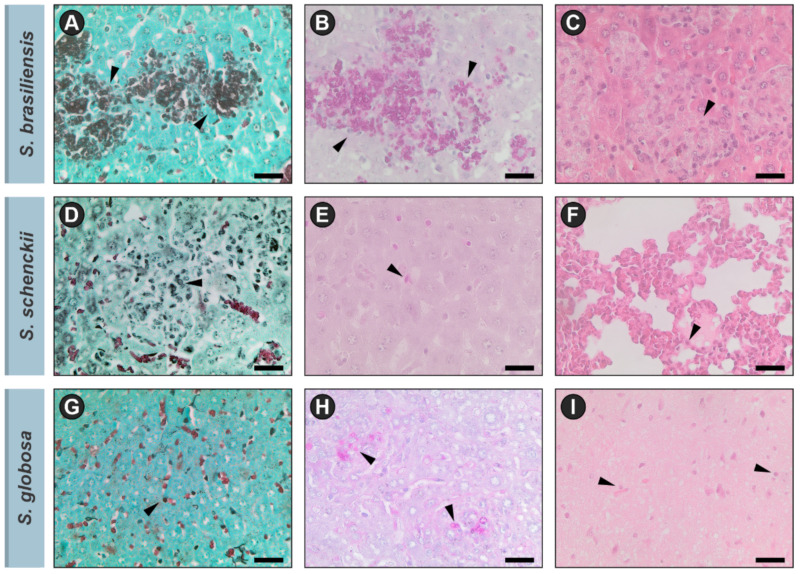
Histopathological patterns of *Sporothrix* species. Black arrows indicate *Sporothrix* yeasts. (**A**–**C**) *S. brasiliensis* stained by GMS, PAS and HE, respectively; (**D**–**F**) *S. schenckii* stained by GMS, PAS and HE, respectively; (**G**–**I**) *S. globosa* stained by GMS, PAS and HE, respectively. GMS: Gomori methenamine silver, PAS: periodic acid–Schiff-stained, HE: hematoxylin and eosin. Bar = 25 µm.

The histological features of primary cutaneous sporotrichosis are a combination of granulomatous and pyogenic reactions. The yeast cells can be observed in tissue by staining with hematoxylin and eosin (HE), Gomori methenamine silver (GMS), or periodic acid–Schiff (PAS) (Figure 6). Although, as well as in the direct examination, the sensitivity of this test is low for humans due to the scarcity of yeast cells, for felines this method allows the visualization of oval or cigar-shaped yeasts in the tissue, sometimes surrounded by eosinophilic material, constituting the asteroid body [1,27,76,77]. Gonsales et al. reported that cell block cytology (imprint) is an efficient, rapid, and sensitive tool for diagnosing sporotrichosis in cats [71].

During interaction with the human [78] or feline host [79], several *Sporothrix* molecules trigger an immune response leading to the production of immunoglobulin G (IgG), IgM, and IgA in sera [80]. Most of the serological assays employed for the diagnosis of sporotrichosis, such as immunoblot [79,81,82,83,84], latex agglutination [85] and ELISA [86,87], were developed to detect circulating antibodies. ELISA tests stand out as they present greater sensitivity and specificity in detecting circulating antigens or antibodies (usually IgG) for humans [86,87] or feline sporotrichosis [88].

Although the culture-based, biochemical, and immunological methods depicted above are still widely used to diagnose sporotrichosis, they have several drawbacks, such as being time-consuming, unspecific, having low sensitivity, and more importantly are unable to speciate *Sporothrix*. To overcome this problem and speed up the diagnosis of sporotrichosis, the molecular methods complement and are gradually replacing the classical mycological assays to quickly and accurately detect and/or differentiate molecular siblings in *Sporothrix* [89,90].

Nevertheless, in cases with negative molecular tests, especially when antifungal therapy is ineffective, it is important to consider other skin diseases that mimic sporotrichosis. Differential diagnosis of cutaneous sporotrichosis includes cutaneous tuberculous and nontuberculous mycobacterial infections, cutaneous leishmaniasis, chromoblastomycosis, leprosy, mycetoma, and squamous cell carcinoma [27].

This review provides information on the molecular diagnosis and genotyping tools applied for emerging sporotrichosis agents in light of recent taxonomic changes.

## 3. Molecular Diagnosis

Molecular techniques consist of methods for detecting biomarkers such as DNA, RNA, and gene products of a microorganism [91]. The development of molecular diagnostic techniques requires scientists to use certain criteria for assay success, for example, minimal sample preparation to avoid contamination [92,93]. The fungal cell wall is a rigid structure that protects the contents of the cell, and therefore its disruption during DNA extraction is a major challenge that often requires a combination of physical (e.g., bead beating) and chemical methods (e.g., enzymatic digestion). However, developments in molecular diagnostics, including commercial kits for DNA extraction and PCR assays, have supported important advances in detection and speciation assays [94]. Judging from external quality assessment schemes, it is recommended that the purity and concentration of the extracted DNA be evaluated spectrophotometrically (260/280 nm), and a ratio of ~1.8 is commonly accepted as “pure” for DNA [95]. Based on our experience with the diagnosis of sporotrichosis, the suitability of DNA samples for the PCR-based assays should be evaluated by amplifying universal markers such as the ITS1/2+5.8S region for fungal DNA obtained from pure cultures [96]; GAPDH gene (chr12) for clinical samples containing human DNA (i.e., fresh or formalin-fixed and paraffin-embedded tissue blocks) [97]; 28S region for clinical samples containing cat DNA [98]; and the β-actin gene for samples containing murine DNA [99]. Samples that generate positive amplification signals are considered free of PCR inhibitors [89,100,101]. Moreover, extensive performance validation data such as reproducibility, specificity, sensibility, accuracy, diagnostic strategy, and time required for identification must be considered during the development of a diagnostic assay [92,93,102,103,104]. A major advantage of molecular tools is detecting DNA from samples that cannot be cultured, facilitating the diagnosis [104].

**Figure 7:**
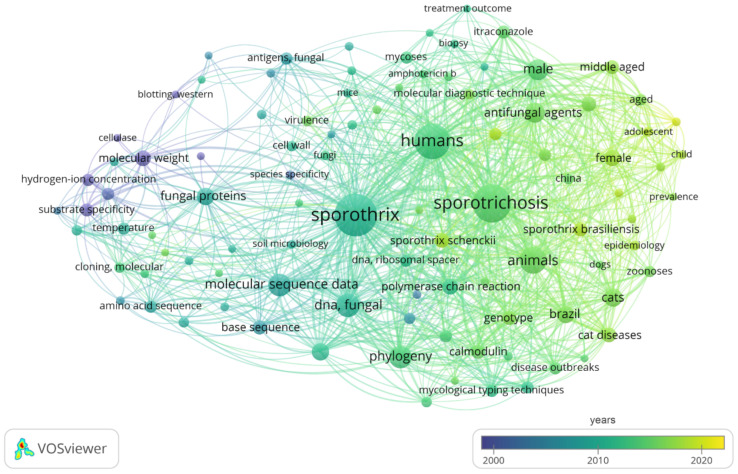
Bibliometric analysis in the molecular diagnostics of sporotrichosis through the software VOSviewer v1.6.13. Network of the 101 keywords co-occurrence found in Pubmed search for molecular diagnosis of sporotrichosis.

Here, the software VOSviewer 1.6.13 was used to explore bibliometric networks and research priorities in the molecular diagnosis of sporotrichosis using the terms “(*Sporothrix* OR Sporotrichosis) AND (molecular diagnosis OR molecular diagnostics OR molecular characterization OR molecular epidemiology)” [105]. We retrieved 200 articles in the PubMed database between 1990 and 2022 (accessed: 6 June 2022) (Figure 7).

In the year-based overlay visualization of the keywords, a total of 101 keywords (frequency ≥5, representing 12.27%) appeared with time information, revealing that research on the molecular diagnosis of sporotrichosis experienced a recent progressive evolution, where only in the last decade were methods capable of genotyping and identifying emerging species such as *S. brasiliensis* and *S. globosa* described [10,13,89,106,107,108,109,110,111,112,113,114,115,116,117,118,119]. Mature keywords such as ‘base sequence’, ‘DNA sequence analysis’, and ‘molecular sequence data’ progressively appeared around the 2000s [120,121,122,123,124,125,126,127,128,129,130,131,132]. DNA sequencing and phylogenetic analyses became more common in the diagnostic scenario from the 2010s onwards [10,13,75,108,110,133]. Therefore, most keywords such as ‘fungal DNA’, ‘polymerase chain reaction’, ‘differential diagnosis’, ‘phylogeny’, ‘genotype’, ‘mycological typing techniques’ and ‘molecular diagnostic techniques’, have emerged only during the past decade [43,49,89,106,107,112,113,115,116,134,135,136,137,138,139,140,141,142,143,144]. Judging from the circle size of the keywords, we found that ‘fungal DNA’, ‘molecular diagnostic techniques’, ‘genotype’, ‘polymerase chain reaction’, and ‘molecular sequence data’ were the main research focuses (Figure 7, Supplementary Table S2).

PCR-based methods emerged as a revolutionary tool in diagnostics due to the ease of generating numerous copies of nucleic acids from isolated strains or directly from clinical samples [93,145,146]. Over the past decades, nearly all techniques available for molecular diagnosis of sporotrichosis involve PCR amplification; however, only a few methods can detect *Sporothrix* DNA directly from clinical samples [100,118,119,122,125,130,147,148,149,150]. The molecular diagnosis of sporotrichosis is currently moving towards detecting pathogen DNA directly from clinical samples in multiplex qPCR assays [89,113].

DNA sequencing is considered the reference method to speciate *Sporothrix*, and ITS is used as a primary barcoding marker [110]. Protein-coding loci are used to speciate *Sporothrix* in addition to exploring genetic diversity, and calmodulin (CAL) [5], β-tubulin (BT2) [4,151], translation elongation factor (EF-1α) [133], and chitin synthase (CHS1) [4,122] stand out among the most used markers.

**Figure 8:**
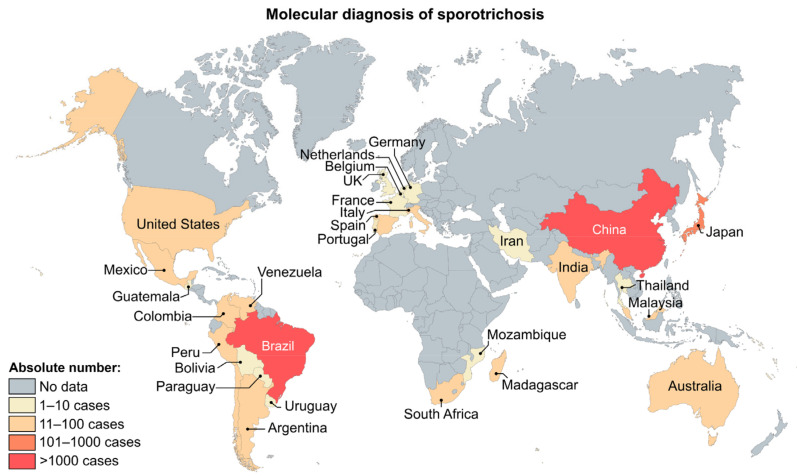
Distribution of sporotrichosis cases diagnosed by molecular assays globally from 2007 to 2020. According to molecular-based characterization, the distribution patterns observed show that Brazil and China are the predominant endemic regions to perform molecular diagnosis of sporotrichosis. References of the molecular epidemiological data for the methodology are available in Supplementary Table S4.

**Figure 9:**
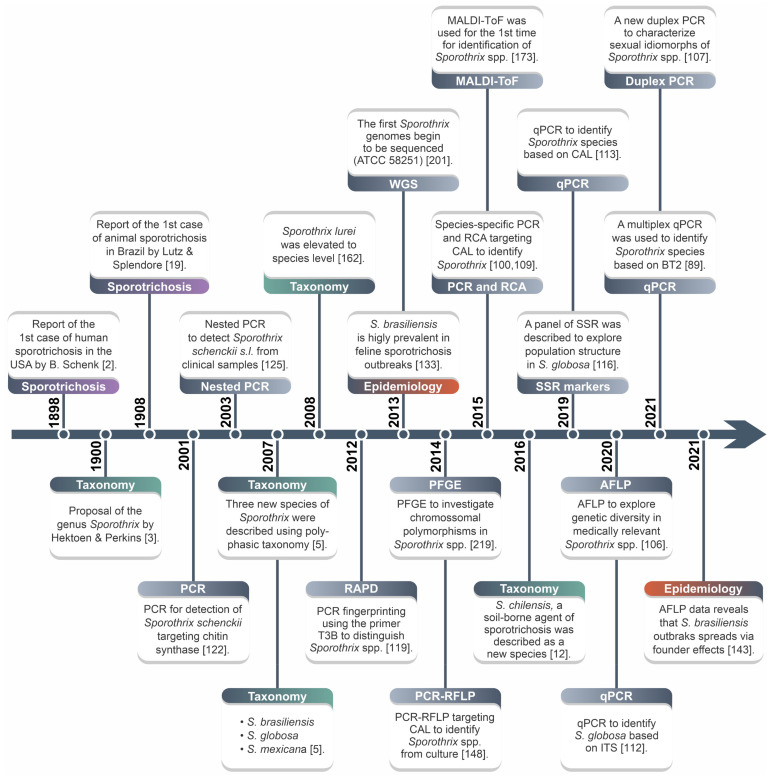
Major developments in the diagnosis/genotyping of the *Sporothrix* species. MLSA: multi-locus sequence analysis; RAPD: randomly amplified polymorphic DNA; CAL: calmodulin; PCR-RFLP: polymerase chain reaction-restriction fragment length polymorphism; WGS: whole-genome sequencing; PFGE: pulsed-field gel electrophoresis; RCA: rolling circle amplification; MALDI-ToF: matrix-assisted laser desorption ionization time-of-flight mass spectrometry; SSR: simple sequence repeat; qPCR: quantitative polymerase chain reaction; AFLP: amplified fragment length polymorphism.

The introduction of molecular methods in routine microbiology laboratory practice to speciate *Sporothrix* impacts surveillance programs, allowing a more accurate assessment of the expansion of ongoing outbreaks. In Figure 8, the distribution of 2394 *Sporothrix* isolates is depicted based on worldwide literature reports based on molecular methods. The search strategy is described in Supplementary Table S3. Below, molecular diagnostic techniques are reviewed (Figure 9).

### 3.1. Internal Transcribed Spacer (ITS)

Internal transcribed spacer (ITS) is a universal barcode marker used for fungal identification [152,153,154,155]. DNA barcoding was first applied in 2003 by Hebert et al. [156], and since then several studies have demonstrated that the polymorphisms in the regions flanking the 5.8S rDNA can be successfully used to distinguish fungal species [152,153,154].

The sequencing of the ITS region is recommended for the molecular diagnosis of *Sporothrix* species, as it is a valuable marker for species-level identification. The low barcoding gap and high copy number are advantageous as they improve robustness and increase detection sensitivity [155,157]. Primers ITS1 and ITS4 or ITS5 and ITS4 are used for amplification and DNA sequencing [96,108]. ITS-based identification has significant discriminatory power over agents embedded in the clinical clade (i.e., *S. brasiliensis*, *S. schenckii*, *S. globosa* and *S. luriei*). On the other hand, for members of the *S. pallida* complex (e.g., *S. chilensis*, *S. mexicana*, *S. humicola* and *S. pallida*), a secondary barcoding marker is required, and the best choice is β-tubulin (BT2) [4,12,151].

**Figure 10:**
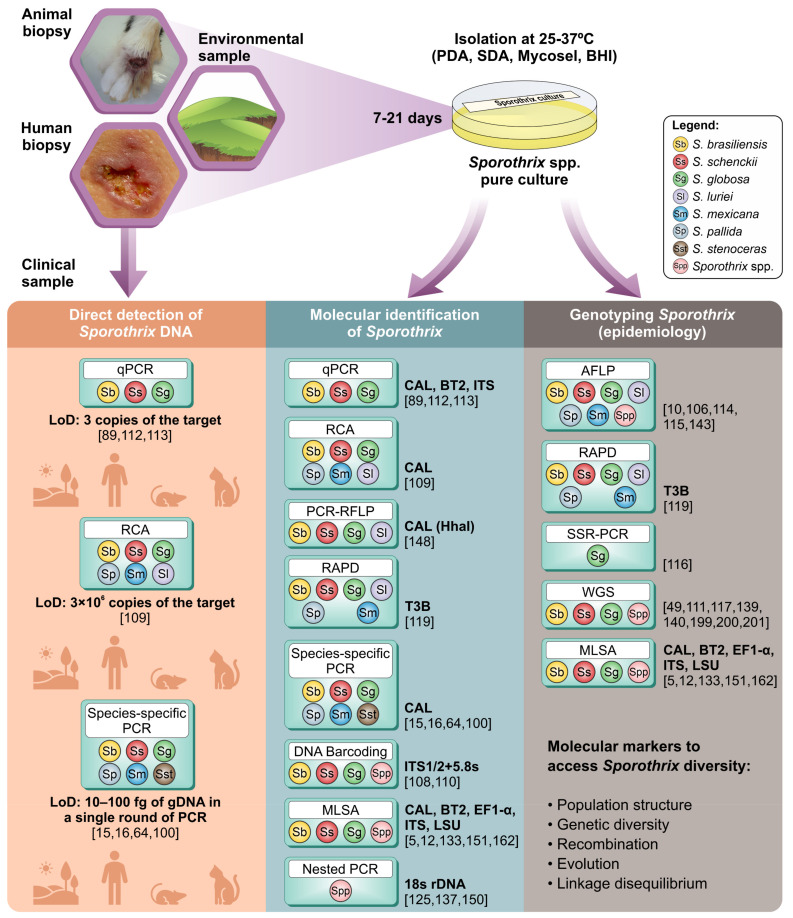
Schematic representation of *Sporothrix* species molecular detection/identification strategies, directly from clinical and/or environmental samples (orange panel) or gDNA extracted from cultured isolates (blue and brown panel). PCR: polymerase chain reaction; PCR-RFLP: polymerase chain reaction-restriction fragment length polymorphism; RCA: rolling circle amplification; qPCR: quantitative polymerase chain reaction; MLSA: multi-locus sequence analysis; AFLP: amplified fragment length polymorphism; SSR: simple sequence repeats; WGS: whole-genome sequencing; RAPD: randomly amplified polymorphic DNA; LoD: limit of detection.

Berbee and Taylor were the first to sequence the 5.8S rDNA region from members of Ophiostomatales. The phylogenetic proximity between *S. schenckii* s.l. and *Sporothrix stenoceras* (formerly *Ophiostoma stenoceras*) supported an anamorph–teleomorph connection [158]. Nevertheless, de Beer et al., using ITS1/2+5.8S sequences, concluded that this historical anamorph–teleomorph connection was erroneous [124], which was later confirmed by Rodrigues et al. using CAL sequences from a large set of clinical and environmental species belonging to the clinical clade and members of the *S. stenoceras* complex [13]. This reinforces the importance of using long sequences covering ITS1/2+5.8S for correct identification. Sequences with poor quality or those shorter than 600 bp should be avoided to identify medically relevant *Sporothrix* (Figure 10).

### 3.2. Multi-Locus Sequence Analysis (MLSA)

Multi-locus sequence analysis (MLSA) is used to infer phylogenetic relationships. Typically, 3–5 housekeeping genes are used as phylogenetic markers, and the sequences are concatenated to assess clustering patterns among strains [159]. The technique is based on multi-locus sequence typing (MLST), a microbial typing method for epidemiological and population genetic structures [160]. MLSA helps increase the taxonomic resolution between *Sporothrix* nested in the clinical and environmental clades.

Loci used for phylogenetic analysis may include a combination of CAL, BT2, EF-1α, CHS1 and ITS1/2+5.8S (Figure 10). The CAL locus (exons 3*–*5) is a good marker to differentiate *Sporothrix* species and has been used in several studies involving this fungus, including the first description of *S. brasiliensis*, *S. globosa*, and *S. mexicana* [4,5,161]. Although CAL can differentiate species, it cannot provide sufficient data about intraspecific genetic diversity for *S. brasiliensis* and *S. globosa* [106]. BT2 [4,8,12], EF-1α [10,12,133], and CHS1 [4,122] are also widely used to ensure the effective identification of sporotrichosis agents. In 2008, Marimon et al. elevated *S. schenckii* var. *luriei* to *S. luriei* using CHS1, BT2, and CAL genes [162]. In 2016, Rodrigues et al. [12] described *S. chilensis*, a new species belonging to the *Sporothrix* pallida complex, sequencing the BT2, CAL, EF-1α, and ITS regions. An excellent example of using multiple gene phylogenies to solve taxonomic questions in *Sporothrix* can be found in the study by de Beer et al. [151], who propose to split *Sporothrix* and *Ophiostoma*. Judging from the studies above, multiple phylogenies are constantly applied in *Sporothrix* taxonomy, although multi-locus sequence analysis is not a frequent practice. We, therefore, recommend using MLSA to boost taxonomic resolution.

### 3.3. Conventional Polymerase Chain Reaction

Conventional polymerase chain reaction (PCR) was first applied in 1985 by Mullis et al. to amplify genetic material, rapidly providing a large amount of specific DNA from an initial template. The technique is based on three steps: DNA denaturation at elevated temperatures (~95 °C), primer annealing, and polymerase extension, which requires the presence of a thermostable DNA polymerase. The process relies on using a thermocycler, equipment capable of switching temperatures according to cycle set, and the results can be analyzed through agarose gel electrophoresis [154,163]. This molecular assay is the most used tool to identify and diagnose sporotrichosis due to the best cost–benefit ratio and faster detection [100].

In 2001, Kano et al. developed a PCR to detect *Sporothrix* DNA directly from tissue samples targeting the CHS1 gene. The lower detection limit of the test was 10 pg of genomic DNA (gDNA) [122,126]. This PCR assay was also used to diagnose feline sporotrichosis [131]. Since 2001, several other studies based on PCR have been developed and applied to detect medically relevant *Sporothrix*; however, these methods do not have significant discriminatory power over sibling species embedded in the clinical clade, thus providing a generic identification [130,147,164]. Rodríguez-Brito et al. [147] evaluated a conventional PCR based on the 18S ribosomal DNA gene from *Sporothrix* species and demonstrated a detection limit of 20 pg to diagnose sporotrichosis. A species-specific PCR was used to identify *S. brasiliensis*, *S. schenckii*, *S. globosa*, *S. mexicana*, *S. pallida*, and *S. stenoceras* using DNA extracted from isolated samples or clinical specimens from experimentally infected BALB/c mice [100]. The species-specific primers were designed to target polymorphisms in the CAL gene, and the detection limit of this assay was 10–100 fg of gDNA. This species-specific PCR assay was successfully used to diagnose feline sporotrichosis [64] (Figure 10). Several studies have applied species-specific PCR to speciate *Sporothrix* [23,40,64,106,142,143,144].

### 3.4. Nested PCR

Nested PCR is a variation of conventional PCR, which uses two primer sets in subsequent reactions to increase the sensitivity and specificity of the reaction. The amplicon generated in the first round of PCR is used as a template for the second round of amplification, using a different pair of primers. Two rounds of PCR make this method more specific than conventional PCR by reducing the nonspecific binding of the products generated in the first PCR [125].

The first description of a nested PCR assay to detect *Sporothrix* was done by Hu et al. [125], and the primers were developed to target the 18S rDNA gene. This region belongs to the fungal nuclear ribosomal operon, which is organized in tandem repeats in the nuclear genome, with several copies per genome, ensuring the high sensitivity of the assay. The method had a detection limit of 40 fg of *Sporothrix* DNA from cultures to biological samples [125]. Recently, this assay was successfully used for the genus-level diagnosis in formalin-fixed and paraffin-embedded (FFPE) samples of cats with sporotrichosis [165]. Interestingly, in experimentally infected mice, Mendoza et al. [166] reported low performance of Hu et al. assay [125] when compared with the diagnosis obtained by culture and direct examination [166].

Xu et al. [150] used a nested PCR assay targeting the 18S rDNA gene to detect *S. schenckii* sensu lato from the tissues of infected mice and skin biopsies of patients with sporotrichosis. A lower detection limit of 50 fg was observed. In 2019, Hayashi et al. [137] investigated the diagnostic value of nested PCR for diagnosing sporotrichosis from FFPE tissues and obtained a remarkable sensitivity of 100% and specificity of 98.7%.

The main drawbacks of the methods above are the inability to speciate molecular siblings in *Sporothrix* and the high chance of amplicon contamination (i.e., false positivity).

### 3.5. Rolling Circle Amplification (RCA)

Rolling circle amplification (RCA) was first described in the 1990s by Fire et al. as a technique capable of rapidly synthesizing multiple copies of circular molecules of DNA or RNA from low concentrations under isothermal conditions [167]. RCA is currently a technique that uses large padlock probes (~100 bp) bearing right and left arms targeting polymorphisms in the sequence of the microorganism of interest, which is very helpful for rapid and accurate diagnostics of infectious diseases [168].

Rodrigues et al. [109] used RCA for species-specific identification of *Sporothrix*. Six padlock probes directed to polymorphisms in the gene encoding calmodulin were developed to speciate *S. brasiliensis*, *S. schenckii*, *S. globosa*, *S. luriei*, *S. mexicana* and *S. pallida*. The method showed specificity and sensitivity of 100% from samples originating from cultures. Moreover, as RCA reactions are carried out using a robust strand-displacing DNA polymerase such as Bst, the method proved to be a useful tool for monitoring the spread of *Sporothrix* in environmental samples (e.g., soil and plants). The method has many advantages: high specificity and sensitivity, facility to perform and interpret results, fast diagnosis, and, most importantly, not requiring special equipment to perform the test (Figure 10).

### 3.6. Quantitative Real-Time PCR (qPCR)

Quantitative real-time PCR (qPCR) is an advanced fluorescence-based method in which a hydrolysis probe or an intercalating dye hybridizes into a double-strand DNA [169]. This technique quantifies the amount of DNA in the sample, and the results are graphically displayed in real-time as the amplification cycles proceed. The technique is a good alternative to DNA sequencing, as it is cheaper and faster to apply, helping to reduce the time to diagnosis [89,91].

The first report of qPCR for detecting *Sporothrix* species was by Rodríguez-Brito et al. [147]. The SYBR Green I-based qPCR assay was developed as a multiplex, using primers SS1/SS2 targeting the 18S ribosomal DNA gene from *Sporothrix* and primers JW11/JW12 targeting the kinetoplast DNA (kDNA) minicircles of *Leishmania*. A melting curve analysis was employed to differentiate *Sporothrix* (Tm = 85.5 °C) and *Leishmania* (Tm = 82.6 °C) amplicons. The lower detection limit was 200 pg of *Sporothrix* DNA from clinical samples.

Later, Zhang et al. [113] developed a multiplex probe-based qPCR method targeting the CAL gene to identify down to species level *S. brasiliensis*, *S. schenckii*, and *S. globosa*. The lower detection limits were 100, 10, and 10 copies for *S. brasiliensis*, *S. schenckii*, and *S. globosa*, respectively, and the sensitivity and specificity reached 100%. Zhang et al. [112] developed a singleplex probe-based qPCR assay based on ITS sequence to identify *S. globosa* from clinical specimens from patients. Sensitivity and specificity were 100%, and the detection limit was 10 fg. Recently, Della Terra et al. [89] standardized a multiplex probe-based qPCR assay to identify *S. brasiliensis*, *S. schenckii*, and *S. globosa* in a single reaction. Polymorphisms in the β-tubulin gene were used to design the probes to identify *Sporothrix* species and demonstrated high specificity (100%) (Figure 10). The qPCR developed by Della Terra et al. [89] was considered effective, fast, accurate, and was 10,000× more sensitive than the species-specific PCR developed by Rodrigues et al. [89,100] and 100–33× more sensitive than the qPCR methods developed by Zhang et al. [112,113]. Under a triplex-probe condition, gDNA’s lower detection limit was 10 fg for *S. schenckii*, 0.1 fg for *S. globosa* and 0.01 fg for *S. brasiliensis*. Therefore, the multiplex qPCR system developed by Della Terra et al. [89] can improve diagnostic capacity in *Sporothrix*-affected areas by assisting local animal health agents or veterinarians, quickly identifying and isolating new cases, potentially benefiting thousands of patients infected each year around the world.

### 3.7. Matrix-Assisted Laser Desorption Ionization Time-of-Flight Mass Spectrometry (MALDI-ToF MS)

The matrix-assisted laser desorption ionization time-of-flight mass spectrometry (MALDI-ToF MS) emerged in the 1990s as a useful tool for identifying and diagnosing microorganisms. The method is based on acquiring a protein fingerprint for an unknown species, which is then compared to species-specific protein patterns from reference spectra libraries [170]. Thus, the identification accuracy relies on the spectra quality and the reliability of the database used [171]. The method has an advantage over other identification techniques, as it can be done directly from the culture within a few minutes, and has been considered a promising method to replace phenotypic identification methods [172].

Oliveira et al. [173] introduced MALDI-ToF MS to promote the accurate identification of *Sporothrix* species. The protocol distinguished *S. brasiliensis*, *S. globosa*, *S. mexicana*, *S. schenckii*, *S. luriei*, and *S. pallida* from isolated cultures. Moreover, MALDI-ToF-based identification matched CAL-sequencing identification, which will shorten the time required to identify *Sporothrix*, accelerating the pace of epidemiologic and diagnostic studies in mycology laboratories [173].

## 4. Genotyping Tools

### 4.1. Restriction Fragment Length Polymorphism and PCR-RFLP

Restriction fragment length polymorphism (RFLP) was first introduced in 1980 by Botstein et al. [174]. The principle of the technique consists of the restriction of the gDNA or mitochondrial DNA (mtDNA) using restriction enzymes, and then the fragments generated are separated according to their molecular size using gel electrophoresis. The method can be combined with PCR (PCR-RFLP), consisting of the selective amplification of the fragments using different combinations of nucleotides in the selective primers. The analysis compares the number and length of the digested fragments resolved by gel electrophoresis [148,174].

Historically, from the late 1980s to the early 2000s, genetic polymorphisms among *Sporothrix* isolates were explored by RFLP of mtDNA, revealing two key groups, named A and B [121,128,175,176,177]. Afterward, groups A and B were divided into 17 and 14 genotypes, respectively, and these genotypes are scattered among isolates from Eurasia, the Americas, Africa, and Australia [178]. Kawasaki et al. [179] suggested using the primer pair 975-8038F and 975-9194R, targeting an intergenic region between ATP9 and COX2 genes of the mtDNA, followed by enzymatic digestion of the amplicons with the restriction enzyme AseI to type *Sporothrix* species. Thus, a re-interpretation of historical data [121,128,175,176,177] under the recent taxonomic developments in *Sporothrix* reveals that classical groups A and B of *S. schenckii* sensu lato classified by RFLP of mtDNA correspond to *S. schenckii* and *S. globosa*, respectively [179,180]. In 2004, Watanabe et al. [129] used RFLP analysis of the ITS region and divided isolates into four clusters showing correlations with their geographical origins.

Rodrigues et al. described a PCR-RFLP targeting the CAL gene and digested with the restriction enzyme HhaI to speciate *S. brasiliensis*, *S. schenckii*, *S. globosa*, and *S. luriei*. The technique was demonstrated to be simple and cost-effective, although it is recommended only for identifying *Sporothrix* from samples isolated in vitro [148]. Montenegro et al. [35] used the PCR-RFLP described by Rodrigues et al. [148] to identify the isolates causing an outbreak in São Paulo and revealed *S. brasiliensis* as the causative agent of sporotrichosis in the state (Figure 10). One major advantage of RFLP and PCR-RFLP is their low cost; however, they are laborious and time-consuming techniques that make them difficult to use in routine laboratories [154].

### 4.2. Molecular Typing by Mating-Type (MAT)

Sexual reproduction in Ascomycetes is often controlled by two unlinked multiallelic loci that encode homeodomain transcription factors or pheromones/pheromone receptors [181]. *Sporothrix* is a heterothallic ascomycete where there is typically a two-form mating-type locus or idiomorph called *MAT1-1* and *MAT1-2* [182,183]. The *MAT1-1* encodes a protein with an α domain, and *MAT1-2* encodes a regulatory protein with a high mobility group (HMG-box), a DNA-binding domain [184]. Therefore, the exclusive presence of one of the two mating-type loci requires the encounter of opposite idiomorphs for sexual reproduction. Although sexual reproduction has never been described in *S. brasiliensis*, *S. schenckii*, and *S. globosa*, this phenomenon cannot be ruled out since many *Sporothrix* can reproduce sexually, generating *Ophiostoma*-like structures, such as ephemeral asci and long-necked ophiostomatoid perithecia through which the ascospores are discharged. Sexually reproducing *Sporothrix* species are scattered across the *S. inflata*, *S. stenoceras*, *S. gossypina*, *S. candida*, and *S. pallida* complexes [151]. Sexual development is extremely important for fungi, as sex generates diversity; therefore, the characterization of the sexual idiomorph distribution in a population is considered an important indicator of reproduction modes.

The first study on the mating type of medically relevant *Sporothrix* species was proposed by Kano et al. [184], which confirmed the existence of the *MAT1-2* genes in *S. globosa*. Afterward, the partial *MAT1-1* locus of *S. schenckii* was characterized [185]. Comparative genomic analysis revealed that medically relevant *Sporothrix* species, including the emerging *S. brasiliensis*, are heterothallic and proposed primers to recognize sexual idiomorphs in *Sporothrix* [186]. Allele frequency distributions showed that the *MAT1-1* to *MAT1-2* ratio was not significantly different from 1:1 for *S. brasiliensis*, *S. schenckii*, and *S. globosa*. Notwithstanding, a single *S. brasiliensis* idiomorph seems successful during cat-transmitted sporotrichosis, leading to a skewed *MAT* distribution in Rio de Janeiro and the Rio Grande do Sul epidemics [107,143,186]. Likewise, for *S. schenckii* from Malaysia, an unbalanced ratio of 1:0 was found, suggesting that a clonal strain is the predominant agent of feline sporotrichosis [187]. A molecular survey investigated the mating-type distribution of *Sporothrix* isolates from Espírito Santo, Brazil, and found the predominance of *MAT1-2* isolates in both species (i.e., *S. brasiliensis* and *S. schenckii*), suggesting that *S. brasiliensis* genotypes during outbreaks in the feline population tend to be clonal, which does not imply the absence of sex but the emergence of a successful genotype [142,188]. Thus, the population structure in *Sporothrix* ranges from paucity to regular sexual recombination, which is likely to be influenced by transmission routes or even a phenomenon of small populations [143,189,190].

Recently, de Carvalho et al. [107] proposed a single-tube duplex PCR assay targeting the α-box protein (*MAT1-1*) and HMG-box (*MAT1-2*) to screen sexual idiomorphs among medically relevant *Sporothrix*. Two strategies were developed, including a conventional PCR, followed by agarose gel electrophoresis allowing a straightforward interpretation based on amplicon size, and an SYBR Green I-based qPCR assay, followed by melting curve analysis. A molecular survey confirmed that the *MAT* allele distribution is an important marker for tracking geographic spread during sporotrichosis outbreaks, determining the population structure, occurrence of sexual reproduction, and facilitating in vivo and in vitro crossing studies [107].

### 4.3. Amplified Fragment Length Polymorphism (AFLP)

Vos et al. [191] first described amplified fragment length polymorphism (AFLP) fingerprinting in 1995. AFLPs are DNA fragments usually in the size range of 50–500 bps, resulting from the digestion of gDNA with one or more restriction enzymes followed by the ligation of oligonucleotide adapters to the fragments generated and amplification of a subset of the fragments by selective PCR. Thus, AFLPs are dominant markers that recognize genetic variations between any two fungal genomes due to (i) a mutation in the restriction site for enzymes, (ii) a mutation in the sequence corresponding to the selective bases during selective amplification, and (iii) a deletion/insertion within the amplified fragment [106]. Major published AFLP applications correspond to microorganisms of the fungal kingdom [143,190,192,193,194,195,196].

The first step in the AFLP protocol is the restriction–ligation reaction. Restriction fragments are generated by combining a rare cutter restriction enzyme (6- to 8-base recognition) and a frequent cutter restriction enzyme (4-base recognition). Under proper conditions, enzyme-specific oligonucleotide adapters (10–30 base pairs) form a double-stranded configuration with ends that anneal to the sticky ends of the respective restriction enzyme sites [197].

**Figure 11:**
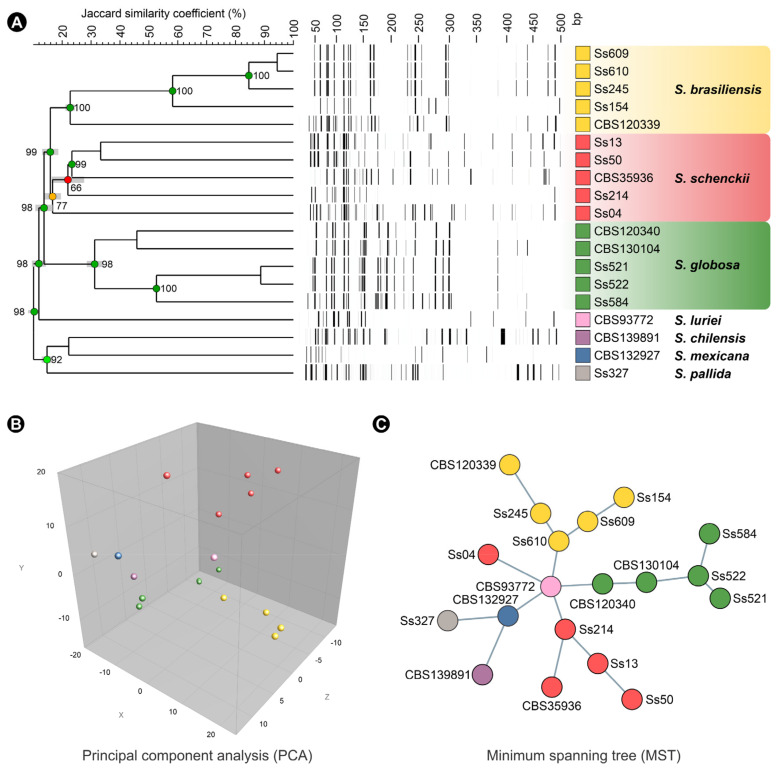
Representation of analyses obtained through AFLP fingerprinting (#5 EcoRI-FAM-GA/MseI-AG) using BioNumerics v.7.6 software. (**A**) Dendrogram, (**B**) principal component analysis (PCA) and (**C**) minimum spanning tree (MST) based on AFLP fingerprint.

Following the restriction–ligation reaction, the next step in the AFLP protocol is the pre-selective amplification aiming to increase the amount of template DNA [196]. Afterward, two selective primers are used for PCR amplification. The first selective primer contains a 5′ section complementary to the adapter and the adjacent rare-cutter restriction site sequence with 3′ selective (1–3 bps) nucleotides extension. The second selective primer also has a 5′ end complementary to the adapter and the frequent-cutter recognition site sequence with an additional 3′ selective nucleotides (1–3 bps) extension [197]. Thus, adjusting the number of selective nucleotides is an essential step toward fingerprints with a manageable number of fragments. AFLP fingerprints may be visualized by classical denaturing polyacrylamide gel electrophoresis using fluorescent or radioactive nucleotides or primers, or by capillary electrophoresis employing fluorescent PCR primers [196]. The relatedness of any two isolates can be investigated through dendrograms, minimum spanning trees (MSTs), or dimensionality reduction methods such as principal components analysis (PCA), as shown in Figure 11.

Neyra et al. [198] were the first to apply the AFLP technique to *S. schenckii* sensu lato in 2005. The Peruvian strains were divided into two clusters unrelated to the geographical origin or clinical form [198]. Subsequently, Zhang et al. applied the technique to *S. brasiliensis*, *S. schenckii*, and *S. globosa*. Diversity was described only in *S. schenckii*, and no genetic diversity was reported for the remaining species. However, the authors describe that *S. brasiliensis* grouped into two different clades, one related to isolates belonging to the Rio Grande do Sul and the other clade comprising isolates belonging to Rio de Janeiro [10]. This AFLP pattern agrees with the studies by Rodrigues et al. [108,133] using DNA sequencing. Zhao et al. [114] described genetic diversity among *S. globosa* isolates using AFLP analysis, contrary to Zhang’s findings [10] describing *S. globosa* as a clonal species. In 2020, Rudramurthy et al. [115] applied AFLP markers to *S. globosa* from India, and they detected low diversity for the 63 isolates used, and there was no correlation between genotypes and clinical presentation or geographic distribution. The main drawback of the above studies is the random choice of selective bases, which may interfere with recognizing cryptic diversity [10,114,115,198].

Judging from this pitfall, de Carvalho et al. [106] took advantage of the growing number of *Sporothrix* genomes available in the NCBI Genome database [111,117,199,200,201,202] combined with extensive in silico analysis [203,204] to develop an effective AFLP scheme, which was later applied to answer questions related to epidemiology, genetic diversity and population structure in *Sporothrix* species. Remarkably, the AFLP scheme (#3 EcoRI-FAM-GA/MseI-TT, #5 EcoRI-FAM-GA/MseI-AG, and #6 EcoRI-FAM-TA/MseI-AA) demonstrated cryptic genetic diversity in species previously thought to be clonal such as *S. brasiliensis* and *S. globosa* [106].

The new AFLP scheme proposed by de Carvalho et al. [106,143] reconstructed the origin, spread, and evolution of the *Sporothrix* outbreaks, describing the recent expansion of *S. brasiliensis* in Brazil, indicating Rio de Janeiro as the epicenter of sporotrichosis. Population genetic analyses revealed for the first time the presence of hybrids of *S. brasiliensis*, *S. schenckii*, and *S. globosa* [143]. Moreover, interpretations of AFLP-based data revealed that the expansion of *S. brasiliensis* occurs through founder effects, a genetic drift phenomenon occurring when a small group of *Sporothrix* in a population splinters off from the parental population and forms a founder population [143] (Figure 10).

Using AFLP markers has several advantages as it does not require prior knowledge of the microorganism’s genome of interest and the ability to access the entire genome in search of polymorphisms at a lower cost than other DNA fingerprint techniques. However, the main limitation of the technique is the numerous steps to reach the result, which can result in errors, especially in the sample manipulations [205].

### 4.4. Simple Sequence Repeats (SSRs)

Microsatellites markers or simple sequence repeats (SSRs) are regions with short tandem repeats (1 to 10 nucleotides) found along prokaryotic or eukaryotic genomes and are widely used in fungal genetics studies, applying both low and high throughput genotyping approaches. Repeat polymorphisms typically result from the addition or deletion of the complete repeat units or motifs triggered by polymerase strand-slippage in DNA replication or recombination errors. Consequently, polymorphisms observed in SSRs for distinct individuals result from differences in the number of repeats of the motifs [206,207].

Microsatellites are codominant markers; therefore, they can discriminate between heterozygotes and homozygotes. SSRs are highly polymorphic, presenting a high information content per gene locus and multi-allelic nature, making the method important to explore diversity in population genetics. DNA sequences flanking SSR markers are usually conserved among individuals of the same species, facilitating the design of species-specific primers to amplify these regions via PCR [116]. SSRs can be classified into four types based on their structure: (i) perfect microsatellites when composed entirely of repeats of a single motif; (ii) imperfect microsatellites when a base pair not belonging to the motif occurs between repeats; (iii) interrupted microsatellites when a sequence of a few base pairs is inserted into the motif; and (iv) composite microsatellites when composed of multiple, adjacent, repetitive motifs [207,208,209].

Gong et al. [116] applied SSRs on *Sporothrix* by developing a panel of 10 microsatellite markers to investigate the diversity in the *S. globosa* population from China. The study suggested that the *S. globosa* population was distributed in three groups, and the genetic variation among the clusters was described [116], corroborating the diversity previously described using AFLP typing [114]. Thus, the usefulness of SSR typing in exploring genetic diversity in *S. globosa* was reported [116] (Figure 10).

Nevertheless, a limitation of SSR markers is that non-*S. globosa* strains were not investigated in the study of Gong et al. [116], thus restricting its application to other medically relevant *Sporothrix* species. Meanwhile, using whole-genomic sequences deposited in public databases can reduce costs and optimize in silico analyses for emerging *Sporothrix* species.

### 4.5. Randomly Amplified Polymorphic DNA (RAPD)

Randomly amplified polymorphic DNA (RAPD) is a technique that employs one or more primers with an arbitrary nucleotide sequence of variable length and is allowed to anneal to the DNA template at low stringency [210]. The amplicons are then resolved electrophoretically to yield DNA fingerprints that differ according to the degree of relatedness of the strains under investigation. A single RAPD marker is not enough to explore genetic diversity in a genome; therefore, a larger number of primers must be employed [211]. Although RAPD has low reproducibility, the technique is useful during epidemiological investigations because it does not require prior knowledge of the genome [212].

Mesa-Arango was the first to perform RAPD in *S. schenckii sensu lato* in 2002 and described distinct patterns that relate to geographical origins without correlation with the clinical form [123]. Liu et al. applied the technique in 2003, highlighting the use of three random primers to investigate DNA polymorphism in *Sporothrix* and demonstrated that the isolates showed different fragment patterns [127]. Reis et al. also performed RAPD using three primers and described polymorphisms in the samples analyzed [212]. In 2012, Oliveira et al. described a PCR fingerprinting using the universal primer T3B to distinguish among *S. brasiliensis*, *S. schenckii*, *S. globosa*, *S. luriei*, *S. mexicana*, and *S. pallida*. The method differentiates each species by different band patterns, describing intraspecific diversity. Moreover, the T3B assay demonstrated 100% agreement with partial calmodulin gene sequencing [119]. Despite exhibiting genetic diversity, these results are subtle compared to other techniques, such as AFLP fingerprinting, which can describe higher intra and interspecific diversity [106]. Other studies applied the PCR fingerprinting using T3B and described good results in identifying species [118,213] (Figure 10).

### 4.6. Pulsed-Field Gel Electrophoresis (PFGE)

Pulsed-field gel electrophoresis (PFGE) is a method of molecular typing used to separate DNA molecules by applying an electric field that changes direction to a gel matrix. The tool was developed in 1984 by Schwartz and Cantor and has a high power of discrimination, being used in epidemiological studies [214]. Among the various applications of the PFGE technique, there are electrophoretic karyotype characterization and physical maps of the genome, identification of similar species, construction of linkage maps and DNA preparation for genome analysis [215,216].

The first PFGE application for *Sporothrix* species was in 1996 by Tateishi et al. In that study, the author defined the karyotypes of *S. schenckii* isolates belonging to Japan; however, the study was performed before the description of the new *Sporothrix* species and it is not clear on which species the study was based [217]. In 2002, O’Reilly et al. demonstrated by PFGE the connection between contact with hay and the increase of cases of sporotrichosis in Western Australia [218].

Chromosome polymorphisms are not a rare event in fungi, and Sasaki et al. [219] revealed through karyotyping the existence of chromosomal polymorphisms, in number and size, among medically relevant *Sporothrix* species. The genetic mapping allowed the identification of syntenic groups, and the hybridization of a chromosomal band of 7.0 Mbp in chromoblot analysis indicates the presence of repeated sequences in the genome, suggesting that recombination occurred in these species. Sasaki et al. significantly contributed to a better understanding of the structure and organization of the genome of *Sporothrix* and were the first to compare the gene mapping among agents of sporotrichosis [219].

### 4.7. Whole-Genome Sequencing (WGS)

Frederick Sanger created a gel-based methodology in the 1970s that coupled a DNA polymerase I with a combination of standard and chain-terminating nucleotides, known as ddNTPs [220], culminating in the “dideoxy” chain-termination method for DNA sequencing. Automated Sanger sequencing is still in use today, mostly in clinical labs where low throughput, higher per-sample costs, and sequencing reads of 500–1000 bp are acceptable.

The second-generation sequencing technologies diverge from Sanger sequencing in several ways, but the key difference is sequencing volume due to multiplexing. In this scenario, a complex library of DNA templates is densely immobilized onto a two-dimensional surface, with all templates accessible to a single reagent volume, making it possible to explore short reads quickly and efficiently. As a result, second-generation sequencing platforms (e.g., Illumina) typically produce reads of ~50–500 bp in length [221,222].

The third-generation sequencing and mapping technologies are currently establishing a new scenario using single-molecule real-time (SMRT) sequencing (e.g., Pacific Biosciences) and nanopore sequencing (e.g., Oxford Nanopore Technologies), generating high-quality genomes. Unlike second-generation, the third-generation technologies generate over 10,000 bp reads or map over 100,000 bp molecules. These long reads allow for the spanning of big structural variants and challenge repetitive regions that confuse short-read sequencers because their short fragments cannot be differentiated from each other during assembly [223,224].

Therefore, the evolution of DNA sequencing over the past 40 years, especially with the introduction of the second- and third-generation sequencing technologies, enables the rapid determination of the sequences of fungal genomes [221,222,225]. From a public health perspective, whole-genome sequencing (WGS) using massively parallel sequencing has become an essential tool for molecular surveillance and epidemiology as it provides the ultimate resolution for tracking sources of disease dissemination, revealing existing variation and its dynamics, the survey of potential drug resistance markers, and genotype-level pathogen incidence monitoring in a high-throughput manner [93,226].

Genomes of *S. brasiliensis*, *S. schenckii*, and *S. globosa*, belonging to the clinical clade, and *S. pallida*, inserted in the environmental clade, have already been sequenced [111,117,200]. The “*S. schenckii* genome project” at the Broad Institute (Cambridge, MA, USA) sequenced the first genome of *S. schenckii* (strain ATCC 58251) using Illumina technology [201]. Afterward, Teixeira et al. [111] provided high-quality genomic sequence assemblies and annotations for *S. schenckii* (ATCC MYA 4821) and *S. brasiliensis* (ATCC MYA 4823) using next-generation 454 pyrosequencing (Roche). Moreover, the comparative genomic analysis revealed a recent habitat shift from a saprobic lifestyle from decaying wood to mammal transmission. In 2016, Huang et al. [117] presented the first genome assemblies of two *S. globosa* strains (CBS 120340 and SS01), providing data to compare the genomes of the three major pathogenic *Sporothrix* species.

In order to understand the emergence of pathogenicity in *Sporothrix*, comparative analyses using members of the environmental clade are imperative. In this scenario, *S. pallida* (strain SPA8) was the first member of the environmental clade that had its complete genome sequenced using Ion Torrent (PGM) (318-chip) and Illumina HiSeq 2000 technologies [200]. An initial investigation revealed that the *S. pallida* genome was approximately 5 Mbp larger than the genomes of its human-pathogenic relatives [200].

To further understand the differences in the pathogenicities of *Sporothrix*, Huang et al. [140] analyzed and annotated the genomes and secondary metabolite biosynthesis of the four main clinical species, along with other rare pathogens and environmental species. Remarkably, the genome size was largest in *S. mexicana* (43.74 Mbp), followed by *S. humicola* (40.74 Mbp), *S. pallida* (40.23 Mbp), *S. inflata* (39.53 Mbp), *S. dimorphospora* (39.13 Mbp), *S. variecibatus* (38.87 Mbp), *S. brunneoviolacea* (37.75 Mbp), *S. luriei* (34.24 Mbp), *S. globosa* (33.29 Mbp), *S. brasiliensis* (33.21 Mbp), and *S. schenckii* (32.23 Mbp). Comparative genomic analysis suggests gene contraction was significant in the evolution of pathogenicity of *Sporothrix* species [140].

New et al. [49] applied WGS to explore genetic variability within *Sporothrix* strains originating from Australia and revealed *S. schenckii* and *S. globosa* as the main agents of human sporotrichosis. Large genetic variations were noted for strains originating from distinct geographic regions. Additionally, phylogenetic analysis based on WGS data provided greater resolution for assessing the relationship between individual isolates [49] compared to the classical CAL marker used in phylogenetic studies [134,227] (Figure 10).

Although a few genomes of *Sporothrix* species have been available in public databases, WGS-based typing tools for public health surveillance and investigation of ongoing epidemics of sporotrichosis are still not a reality in low- and middle-income countries. To investigate population-level diversity, the main hurdle to overcome is the creation of a robust genome-wide SNPs panel allowing for a reliable interchange and comparison of independent datasets among different laboratories from *Sporothrix*-affected areas.

## 5. Perspectives of Future Molecular Methods

Major advances have been made over the years in diagnosing sporotrichosis using molecular assays. *Sporothrix*-directed PCRs have been powerful tools in diagnosing human and animal sporotrichosis during the past decade. Methods developed before recognizing and introducing molecular siblings in *Sporothrix* in 2007 [5] may provide a generic identification. Currently, technologies such as conventional species-specific PCR have contributed to the correct identification down to the species level. They have been one of the most effective techniques due to the ease of performing, the well-established protocol and the ease of diagnosing sporotrichosis directly from clinical specimens, with a detection limit of 10–100 fg in a single PCR reaction [100]. Despite high sensitivity and specificity, molecular techniques are still expensive and may require laboratory infrastructure, which may be prohibitive in low-resource settings, especially if we consider that sporotrichosis repeatedly affects the poorest populations [228,229].

Although molecular techniques are great allies in diagnosing sporotrichosis, most protocols have been applied to DNA obtained from pure cultures, which in the routine laboratory can delay the diagnosis due to the time taken to cultivate the fungus [229,230]. Thus, the scarcity of protocols based on detecting *Sporothrix* DNA directly from clinical samples is an important gap to be filled in molecular diagnosis. Major samples may include pus, exudates, aspirates, and fresh or FFPE tissues. Encouraging results have been published in the past decade using specimens from human, murine or feline origins [64,89,100,112,113,147,165]. Trends in molecular diagnosis of sporotrichosis show that multiplexing is the leading strategy for the near future, allowing the detection and speciation of different agents in real-time reactions [89,113].

From an epidemiological perspective, genotyping is key to tracing back sources of *Sporothrix* infections, which provides essential information to policymakers regarding outbreak management. The principle of all typing schemes presented is that *Sporothrix* isolated from an epidemiological cluster arises from a typical ancestral strain, and therefore, these strains will share characteristics that distinguish them from epidemiologically unrelated strains of the same species. From high-to-low throughput genotyping, MLSA, AFLP, SSR, RAPD, and PCR-RFLP are available to assess the transmission dynamics and sporotrichosis expansion. The use of next-generation sequencing-based strategies, when widely available, will further improve the sensitivity of detection with the potential to increase the resolution of molecular epidemiology studies and, most importantly, patient care in *Sporothrix*-affected areas.

## Data Availability

The data presented in this study are available within the article and Supplementary Materials.

## Supplementary Materials

The following supporting information can be downloaded at: https://www.mdpi.com/article/10.3390/jof8080809/s1. Supplementary Table S1: Strains, species, origin, and GenBank accession numbers of LSU, ITS, BT2 and CAL of *Sporothrix* spp. isolates used in this study to construct the phylogenetic tree. Supplementary Table S2: Summary of keywords used in the bibliometric analysis performed in the software VOSviewer 1.6.13. Supplementary Table S3: Search strategy. Supplementary Table S4: Summary of molecular diagnosis methods in sporotrichosis from 2007 to 2021.
