# Supplementary material for: Trends in Molecular Diagnostics and Genotyping Tools Applied for Emerging Sporothrix Species
Source: J Fungi (Basel). 2022 Jul 31;8(8):809. doi: 10.3390/jof8080809 (PMC9409836; doi:10.3390/jof8080809)
Supplement: Supplementary file 1 [file jof-08-00809-s001.zip › jof-1788096-supplementary.pdf]

Supplementary material

Supplementary material: Trends in molecular diagnostics and genotyping tools applied for emerging *Sporothrix* species

Jamile Ambrósio de Carvalho [1], Ruan Campos Monteiro [1], Ferry Hagen [2,3,4], Zoilo Pires de Camargo [1,5], Anderson Messias Rodrigues [1,5*]

[1] Laboratory of Emerging Fungal Pathogens, Department of Microbiology, Immunology, and Parasitology, Discipline of Cellular Biology, Federal University of São Paulo (UNIFESP), São Paulo 04023062, Brazil.

[2] Department of Medical Mycology, Westerdijk Fungal Biodiversity Institute, Uppsalalaan 8, 3584CT, Utrecht, The Netherlands.

[3] Institute for Biodiversity and Ecosystem Dynamics (IBED), University of Amsterdam, Sciencepark 904, 1098 XH, Amsterdam, The Netherlands.

[4] Department of Medical Microbiology, University Medical Center Utrecht, Heidelberglaan 100, 3584 CX, Utrecht, The Netherlands.

[5] Department of Medicine, Discipline of Infectious Diseases, Federal University of São Paulo (UNIFESP), São Paulo 04023062, Brazil.

* Correspondence: amrodrigues@unifesp.br; Tel.: +55 1155764551 (ext. 1540)

Supplementary Table S1: Strains, species, origin, and GenBank accession numbers of LSU, ITS, BT2 and CAL of *Sporothrix* spp. isolates used in this study to construct the phylogenetic tree.

Strains	Species	Origin	LSU	ITS	BT2	CAL
CBS 121961	*S. variecibatus*	South Africa	DQ821537	DQ821568	DQ821539	KX590813
CBS 118848	*S. stylites*	South Africa	EF139115	EF127883	EF139096	KX590812
CBS 237.32	*S. stenoceras*	Norway	DQ294350	AF484462	DQ296074	JQ511956
CBS 359.36	*S. schenckii*	USA	KX590890	KX590842	AM116911	AM117437
CBS 116.78	*S. rossii*	USA	KX590844	KX590815	KX590754	JQ511972
CBS 251.88	*S. prolifera*	Poland	KX590869	KX590829	KX590770	KX590797
CBS 669.88	*S. polyporicola*	Sweden	KX590866	KX590827	KX590768	KX590796
CBS 119721	*S. phasma*	South Africa	DQ316151	DQ316219	DQ316181	KX590795
CBS 119590	*S. palmiculminata*	South Africa	DQ316143	DQ316191	DQ316153	KX590794
CBS 131.56	*S. pallida*	Japan	EF139121	EF127880	EF139110	KX590811
CBS 138.5	*S. narcissi*	Netherlands	KX590861	AY194510	KX590765	KX590791
CBS 120341	*S. mexicana*	Mexico	KX590887	KX590841	AM498344	AM398393
CBS 937.72	*S. luriei*	South Africa	KX590886	AB128012	AM747289	AM747302
CBS 112927	*S. lunata*	Austria	KX590859	AY280485	AY280466	JQ511970
CBS 118129	*S. humicola*	South Africa	EF139114	AF484472	EF139100	KX590808
CBS 437.76	*S. gutturiformis*	Malaysia	KX590885	KX590839	KX590778	KX590807
ATCC18999	*S. gossypina*	USA	KX590856	KX590819	KX590761	KX590789
CBS 120340	*S. globosa*	Spain	KX590884	KX590838	AM116966	KP101459
CBS 112912	*S. fusiformis*	Azerbaijan	DQ294354	AY280481	AY280461	JQ511967
CBS 424.77	*S. eucastanea*	USA	KX590843	KX590814	KX590753	KX590781
CBS 455.83	*S. dombeyi*	Chile	KX590865	KX590826	KX590767	KX590793
CBS 115790	*S. dentifunda*	Hungary	KX590853	AY495434	AY495445	KX590787
CBS 129713	*S. candida*	South Africa	KX590850	HM051409	HM041874	KX590785
CIEFAP456	*S. cabralii*	Argentina	KT362229	KT362256	KT381295	KX590804
CBS 124561	*S. brunneoviolacea*	Spain	KX590878	FN546959	FN547385	KX590803
CBS 120339	*S. brasiliensis*	Brazil	KX590877	KX590832	AM116946	AM116899
CBS 474.91	*S. bragantina*	Brazil	KX590849	FN546965	FN547387	KX590784
CBS 118837	*S. aurorae*	South Africa	KX590848	DQ396796	DQ396800	KX590783
CBS 125.89	*S. abietina*	Mexico	KX590845	AF484453	KX590755	JQ511966

LSU: Large subunit; ITS: Internal Transcribed Spacer (ITS1/ITS2+5.8S); BT2: β-tubulin; CAL: Calmodulin (exons 3–5). Sequences reference: [1]

Supplementary Table S2: Summary of keywords used in the bibliometric analysis performed in the software VOSviewer 1.6.13.

Keyword	Occurrences	Total Link Strength
Sporothrix	147	1135
Sporotrichosis	122	949
Humans	102	837
Animals	61	521
Phylogeny	43	416
DNA, fungal	45	412
Male	36	372
Molecular sequence data	42	372
Male	36	370
Antifungal agents	32	308
Cats	30	299
Brazil	27	273
Female	24	267
Sequence analysis, DNA	26	265
Adult	18	225
Calmodulin	18	221
Polymerase chain reaction	21	218
Calmodulin	17	214
Fungal proteins	26	211
Cat diseases	20	207
Genotype	17	200
Middle aged	17	197
Sporothrix schenckii	18	176
Microbial sensitivity tests	13	164
Sporothrix brasiliensis	18	153
Aged	11	147
Disease outbreaks	12	142
Mycological typing techniques	13	141
Base sequence	16	140
Genetic variation	12	132
Itraconazole	14	131
Zoonoses	11	131
DNA, ribosomal spacer	13	130
Adolescent	8	121
Molecular epidemiology	12	121
Molecular weight	21	119
Child	7	109
Child, preschool	7	109
Cluster analysis	9	107
Sporothrix globosa	13	107
Amino acid sequence	12	102

Keyword	Occurrences	Total Link Strength
Virulence	10	100
Young adult	7	100
Molecular diagnostic techniques	10	99
China	11	86
Cloning, molecular	10	86
Polymorphism, restriction fragment length	11	86
Temperature	12	81
Antigens, fungal	12	80
Sequence alignment	9	80
Epidemiology	8	79
Substrate specificity	14	79
Amphotericin B	7	77
Hydrogen-ion concentration	12	75
Enzyme stability	9	73
Genome, fungal	6	73
Cell wall	9	71
Kinetics	12	69
Molecular typing	5	68
Sensitivity and specificity	8	67
Biopsy	7	66
Dogs	5	66
Mycoses	13	65
Sequence homology, amino acid	7	64
Molecular identification	6	62
Mice	6	61
Prevalence	5	61
DNA fingerprinting	6	60
DNA, ribosomal	5	60
Species specificity	7	60
Mice, inbred BALB c	6	59
Skin	6	59
Recombinant proteins	7	57
Phenotype	5	56
Disease models, animal	6	54
Histoplasma	8	54
Terbinafine	5	54
Gene expression	6	53
DNA, mitochondrial	7	52
Genes, fungal	6	52
Antibodies, fungal	7	51
Treatment outcome	5	50
Gene expression regulation, fungal	6	47
Mycelium	5	47

Keyword	Occurrences	Total Link Strength
Soil microbiology	6	47
Microbiological techniques	5	44
Retrospective studies	5	44
Endemic diseases	6	42
Fungi	8	41
Dermatomycoses	5	38
Diagnosis, differential	5	38
Saccharomyces cerevisiae	5	37
Candida albicans	5	35
Glycosylation	5	35
Chromatography, gel	5	33
Histoplasmosis	6	33
South africa	5	33
Blotting, western	5	29
Paracoccidioides	5	29
Paracoccidioidomycosis	5	24
Oligosaccharides	5	19
Cellulase	5	17

Supplementary Table S3: Search strategy.

Search #	Terms	Period covered	N. of articles
1	*Sporothrix* AND molecular diagnosis	2007-2021	52
2	*Sporothrix* AND molecular diagnostics	2007-2021	39
3	*Sporothrix* AND molecular epidemiology	2007-2021	54
4	Sporotrichosis AND molecular diagnosis	2007-2021	59
5	Sporotrichosis AND molecular diagnostics	2007-2021	39
6	Sporotrichosis AND molecular epidemiology	2007-2021	54

A PubMed search was performed in January 2022. The search terms used were: (#1) *Sporothrix* AND molecular diagnosis; (#2) *Sporothrix* AND molecular diagnostics; (#3) *Sporothrix* AND molecular epidemiology; (#4) Sporotrichosis AND molecular diagnosis; (#5) Sporotrichosis AND molecular diagnostics; (#6) Sporotrichosis AND molecular epidemiology, and 53-39-54-60-40-54 results were returned, respectively. These results were manually filtered to recover articles that describe *Sporothrix* spp. using molecular methods from 2007 to 2021.

Supplementary Table S4: Summary of molecular diagnosis methods in sporotrichosis from 2007 to 2021.

Methodology	Year	Reference
RFLP	2007	[2]
Sequencing	2007	[3]
RFLP	2007	[4]
Sequencing	2008	[5]
PCR	2008	[6]
Sequencing	2008	[7]
Sequencing	2008	[8]
RAPD	2009	[9]
Sequencing	2010	[10]
Sequencing	2010	[11]
Sequencing	2011	[12]
Sequencing	2011	[13]
Sequencing	2011	[14]
Sequencing	2012	[15]
Sequencing	2012	[16]
Sequencing	2013	[17]
Sequencing	2013	[18]
Sequencing	2013	[19]
Sequencing	2014	[20]
RFLP	2014	[21]
Sequencing	2014	[22]
PCR-RFLP	2014	[23]
Sequencing	2014	[24]
Sequencing	2014	[25]
Sequencing	2014	[26]
Sequencing	2014	[27]
Sequencing	2014	[28]
PCR	2015	[29]
Sequencing	2015	[30]
Sequencing	2015	[31]
Sequencing	2015	[32]
Sequencing	2015	[33]
Sequencing	2015	[34]
Sequencing	2015	[35]
Sequencing	2015	[36]
PCR-RFLP	2015	[37]
Species-specific PCR	2015	[38]
Sequencing	2016	[39]
Sequencing	2016	[40]
Sequencing	2016	[41]
Sequencing	2017	[42]

Methodology	Year	Reference
Sequencing	2017	[43]
PCR	2017	[44]
Sequencing	2017	[45]
WGS	2018	[46]
PCR	2018	[47]
Sequencing	2018	[48]
Species-specific PCR	2018	[49]
Sequencing	2018	[50]
Sequencing	2018	[51]
Sequencing	2019	[52]
Sequencing	2019	[53]
Sequencing	2019	[54]
qPCR	2019	[55]
Sequencing	2019	[56]
Sequencing	2019	[57]
Sequencing	2019	[58]
Sequencing	2019	[59]
Sequencing	2019	[60]
Sequencing	2020	[61]
Specific-species PCR	2020	[62]
Sequencing	2020	[63]
Sequencing	2020	[64]
Species-specific PCR	2020	[65]
Species-specific PCR	2020	[66]
qPCR	2020	[67]
Sequencing	2021	[68]
Species-specific PCR	2021	[69]
Sequencing	2021	[70]
Species-specific PCR	2021	[71]
Species-specific PCR	2021	[72]

References

1. de Beer, Z.W.; Duong, T.A.; Wingfield, M.J. The divorce of *Sporothrix* and *Ophiostoma*: solution to a problematic relationship. *Stud Mycol* **2016**, *83*, 165-191, doi:10.1016/j.simyco.2016.07.001.
2. Arenas, R.; Miller, D.; Campos-Macias, P. Epidemiological data and molecular characterization (mtDNA) of *Sporothrix schenckii* in 13 cases from Mexico. *Int J Dermatol* **2007**, *46*, 177-179, doi:10.1111/j.1365-4632.2006.03036.x.
3. Marimon, R.; Cano, J.; Gené, J.; Sutton, D.A.; Kawasaki, M.; Guarro, J. *Sporothrix brasiliensis*, *S. globosa*, and *S. mexicana*, three new *Sporothrix* species of clinical interest. *J Clin Microbiol* **2007**, *45*, 3198-3206, doi:10.1128/JCM.00808-07.
4. Fujii, H.; Tanioka, M.; Yonezawa, M.; Arakawa, A.; Matsumura, Y.; Kore-eda, S.; Miyachi, Y.; Tanaka, S.; Mochizuki, T. A case of atypical sporotrichosis with multifocal cutaneous ulcers. *Clin Exp Dermatol* **2008**, *33*, 135-138, doi:10.1111/j.1365-2230.2007.02572.x.
5. Galhardo, M.C.; De Oliveira, R.M.; Valle, A.C.; Paes Rde, A.; Silvatavares, P.M.; Monzon, A.; Mellado, E.; Rodriguez-Tudela, J.L.; Cuenca-Estrella, M. Molecular epidemiology and antifungal susceptibility patterns of *Sporothrix schenckii* isolates from a cat-transmitted epidemic of sporotrichosis in Rio de Janeiro, Brazil. *Med Mycol* **2008**, *46*, 141-151, doi:10.1080/13693780701742399.
6. Criseo, G.; Malara, G.; Romeo, O.; Puglisi Guerra, A. Lymphocutaneous sporotrichosis in an immunocompetent patient: a case report from extreme southern Italy. *Mycopathologia* **2008**, *166*, 159-162, doi:10.1007/s11046-008-9121-4.
7. de Meyer, E.M.; de Beer, Z.W.; Summerbell, R.C.; Moharram, A.M.; de Hoog, G.S.; Vismer, H.F.; Wingfield, M.J. Taxonomy and phylogeny of new wood- and soil-inhabiting *Sporothrix* species in the *Ophiostoma stenoceras-Sporothrix schenckii* complex. *Mycologia* **2008**, *100*, 647-661, doi:10.3852/07-157r.
8. Marimon, R.; Gené, J.; Cano, J.; Guarro, J. *Sporothrix luriei*: a rare fungus from clinical origin. *Med Mycol* **2008**, *46*, 621-625, doi:10.1080/13693780801992837.
9. Reis, R.S.; Almeida-Paes, R.; Muniz Mde, M.; Tavares, P.M.; Monteiro, P.C.; Schubach, T.M.; Gutierrez-Galhardo, M.C.; Zancopé-Oliveira, R.M. Molecular characterisation of *Sporothrix schenckii* isolates from humans and cats involved in the sporotrichosis epidemic in Rio de Janeiro, Brazil. *Mem Inst Oswaldo Cruz* **2009**, *104*, 769-774, doi:10.1590/s0074-02762009000500018.
10. Criseo, G.; Romeo, O. Ribosomal DNA sequencing and phylogenetic analysis of environmental *Sporothrix schenckii* strains: comparison with clinical isolates. *Mycopathologia* **2010**, *169*, 351-358, doi:10.1007/s11046-010-9274-9.
11. de Oliveira, M.M.; de Almeida-Paes, R.; de Medeiros Muniz, M.; de Lima Barros, M.B.; Galhardo, M.C.; Zancope-Oliveira, R.M. Sporotrichosis caused by *Sporothrix globosa* in Rio De Janeiro, Brazil: case report. *Mycopathologia* **2010**, *169*, 359-363, doi:10.1007/s11046-010-9276-7.
12. Romeo, O.; Scordino, F.; Criseo, G. New insight into molecular phylogeny and epidemiology of *Sporothrix schenckii* species complex based on calmodulin-encoding gene analysis of Italian isolates. *Mycopathologia* **2011**, *172*, 179-186, doi:10.1007/s11046-011-9420-z.
13. Oliveira, M.M.; Almeida-Paes, R.; Muniz, M.M.; Gutierrez-Galhardo, M.C.; Zancope-Oliveira, R.M. Phenotypic and molecular identification of *Sporothrix* isolates from an epidemic area of sporotrichosis in Brazil. *Mycopathologia* **2011**, *172*, 257-267, doi:10.1007/s11046-011-9437-3.
14. Dias, N.M.; Oliveira, M.M.; Santos, C.; Zancope-Oliveira, R.M.; Lima, N. Sporotrichosis caused by *Sporothrix mexicana*, Portugal. *Emerg Infect Dis* **2011**, *17*, 1975-1976, doi:10.3201/eid1710.110737.
15. Silva-Vergara, M.L.; de Camargo, Z.P.; Silva, P.F.; Abdalla, M.R.; Sgarbieri, R.N.; Rodrigues, A.M.; dos Santos, K.C.; Barata, C.H.; Ferreira-Paim, K. Disseminated *Sporothrix brasiliensis* infection with endocardial and ocular involvement in an HIV-infected patient. *Am J Trop Med Hyg* **2012**, *86*, 477-480, doi:10.4269/ajtmh.2012.11-0441.
16. de Oliveira, M.M.E.; Sampaio, P.; Almeida-Paes, R.; Pais, C.; Gutierrez-Galhardo, M.C.; Zancope-Oliveira, R.M. Rapid identification of *Sporothrix* species by T3B fingerprinting. *J Clin Microbiol* **2012**, *50*, 2159-2162,

doi:10.1128/JCM.00450-12.

17. Rodrigues, A.M.; de Hoog, S.; de Camargo, Z.P. Emergence of pathogenicity in the *Sporothrix schenckii* complex. *Med Mycol* **2013**, *51*, 405-412, doi:10.3109/13693786.2012.719648.
18. Rodrigues, A.M.; de Melo Teixeira, M.; de Hoog, G.S.; Schubach, T.M.P.; Pereira, S.A.; Fernandes, G.F.; Bezerra, L.M.L.; Felipe, M.S.; de Camargo, Z.P. Phylogenetic analysis reveals a high prevalence of *Sporothrix brasiliensis* in feline sporotrichosis outbreaks. *PLoS Negl Trop Dis* **2013**, *7*, e2281, doi:10.1371/journal.pntd.0002281.
19. Oliveira, M.M.; Maifrede, S.B.; Ribeiro, M.A.; Zancope-Oliveira, R.M. Molecular identification of *Sporothrix* species involved in the first familial outbreak of sporotrichosis in the state of Espirito Santo, Southeastern Brazil. *Mem Inst Oswaldo Cruz* **2013**, *108*, 936-938, doi:10.1590/0074-0276130239.
20. Ottonelli Stopiglia, C.D.; Magagnin, C.M.; Castrillon, M.R.; Mendes, S.D.; Heidrich, D.; Valente, P.; Scroferneker, M.L. Antifungal susceptibilities and identification of species of the *Sporothrix schenckii* complex isolated in Brazil. *Med Mycol* **2014**, *52*, 56-64, doi:10.3109/13693786.2013.818726.
21. Montenegro, H.; Rodrigues, A.M.; Galvão Dias, M.A.; da Silva, E.A.; Bernardi, F.; Camargo, Z.P. Feline sporotrichosis due to *Sporothrix brasiliensis*: an emerging animal infection in São Paulo, Brazil. *BMC Vet Res* **2014**, *10*, 269, doi:10.1186/s12917-014-0269-5.
22. Liu, T.-t.; Zhang, K.; Zhou, X. Molecular identification of *Sporothrix* clinical isolates in China. *J Zhejiang Univ Sci B* **2014**, *15*, 100-108, doi:10.1631/jzus.B1300136.
23. Rodrigues, A.M.; de Hoog, G.S.; Camargo, Z.P. Genotyping species of the *Sporothrix schenckii* complex by PCR-RFLP of calmodulin. *Diagn Microbiol Infect Dis* **2014**, *78*, 383–387, doi:10.1016/j.diagmicrobio.2014.01.004.
24. Rodrigues, A.M.; Bagagli, E.; de Camargo, Z.P.; Bosco, S.M.G. *Sporothrix schenckii sensu stricto* isolated from soil in an armadillo's burrow. *Mycopathologia* **2014**, *177*, 199-206, doi:10.1007/s11046-014-9734-8.
25. Estrada-Barcenas, D.A.; Vite-Garin, T.; Navarro-Barranco, H.; de la Torre-Arciniega, R.; Perez-Mejia, A.; Rodriguez-Arellanes, G.; Ramirez, J.A.; Humberto Sahaza, J.; Taylor, M.L.; Toriello, C. Genetic diversity of *Histoplasma* and *Sporothrix* complexes based on sequences of their ITS1-5.8S-ITS2 regions from the BOLD System. *Rev Iberoam Micol* **2014**, *31*, 90-94, doi:10.1016/j.riam.2013.10.003.
26. de Oliveira, M.M.; Verissimo, C.; Sabino, R.; Aranha, J.; Zancope-Oliveira, R.M.; Sampaio, P.; Pais, C. First autochthone case of sporotrichosis by *Sporothrix globosa* in Portugal. *Diagn Microbiol Infect Dis* **2014**, *78*, 388-390, doi:10.1016/j.diagmicrobio.2013.08.023.
27. Sasaki, A.A.; Fernandes, G.F.; Rodrigues, A.M.; Lima, F.M.; Marini, M.M.; dos S. Feitosa, L.; de Melo Teixeira, M.; Felipe, M.S.S.; da Silveira, J.F.; de Camargo, Z.P. Chromosomal polymorphism in the *Sporothrix schenckii* complex. *PLoS ONE* **2014**, *9*, e86819, doi:10.1371/journal.pone.0086819.
28. Rodrigues, A.M.; de Hoog, G.S.; de Cassia Pires, D.; Brilhante, R.S.N.; da Costa Sidrim, J.J.; Gadelha, M.F.; Colombo, A.L.; de Camargo, Z.P. Genetic diversity and antifungal susceptibility profiles in causative agents of sporotrichosis. *BMC Infect Dis* **2014**, *14*, 219, doi:10.1186/1471-2334-14-219.
29. Rodriguez-Brito, S.; Camacho, E.; Mendoza, M.; Nino-Vega, G.A. Differential identification of *Sporothrix* spp. and *Leishmania* spp. by conventional PCR and qPCR in multiplex format. *Med Mycol* **2015**, *53*, 22-27, doi:10.1093/mmy/myu065.
30. Govender, N.P.; Maphanga, T.G.; Zulu, T.G.; Patel, J.; Walaza, S.; Jacobs, C.; Ebonwu, J.I.; Ntuli, S.; Naicker, S.D.; Thomas, J. An outbreak of lymphocutaneous sporotrichosis among mine-workers in South Africa. *PLoS Negl Trop Dis* **2015**, *9*, e0004096, doi:10.1371/journal.pntd.0004096.
31. Borba-Santos, L.P.; Rodrigues, A.M.; Gagini, T.B.; Fernandes, G.F.; Castro, R.; de Camargo, Z.P.; Nucci, M.; Lopes-Bezerra, L.M.; Ishida, K.; Rozental, S. Susceptibility of *Sporothrix brasiliensis* isolates to amphotericin B, azoles, and terbinafine. *Med Mycol* **2015**, *53*, 178-188, doi:10.1093/mmy/myu056.
32. Zhang, Y.; Hagen, F.; Stielow, B.; Rodrigues, A.M.; Samerpitak, K.; Zhou, X.; Feng, P.; Yang, L.; Chen, M.; Deng, S.; et al. Phylogeography and evolutionary patterns in *Sporothrix* spanning more than 14,000 human and animal case reports. *Persoonia* **2015**, *35*, 1-20, doi:10.3767/003158515x687416.
33. Oliveira, M.M.; Franco-Duarte, R.; Romeo, O.; Pais, C.; Criseo, G.; Sampaio, P.; Zancope-Oliveira, R.M.

Evaluation of T3B fingerprinting for identification of clinical and environmental *Sporothrix* species. *FEMS Microbiol Lett* **2015**, *362*, doi:10.1093/femsle/fnv027.

34. Camacho, E.; León-Navarro, I.; Rodríguez-Brito, S.; Mendoza, M.; Niño-Vega, G.A. Molecular epidemiology of human sporotrichosis in Venezuela reveals high frequency of *Sporothrix globosa*. *BMC Infect Dis* **2015**, *15*, 94, doi:10.1186/s12879-015-0839-6.
35. Kano, R.; Okubo, M.; Siew, H.H.; Kamata, H.; Hasegawa, A. Molecular typing of *Sporothrix schenckii* isolates from cats in Malaysia. *Mycoses* **2015**, *58*, 220-224, doi:10.1111/myc.12302.
36. de Araujo, M.L.; Rodrigues, A.M.; Fernandes, G.F.; de Camargo, Z.P.; de Hoog, G.S. Human sporotrichosis beyond the epidemic front reveals classical transmission types in Espírito Santo, Brazil. *Mycoses* **2015**, *58*, 485-490, doi:10.1111/myc.12346.
37. Sanchotene, K.O.; Madrid, I.M.; Klafke, G.B.; Bergamashi, M.; Terra, P.P.D.; Rodrigues, A.M.; de Camargo, Z.P.; Xavier, M.O. *Sporothrix brasiliensis* outbreaks and the rapid emergence of feline sporotrichosis. *Mycoses* **2015**, *58*, 652-658, doi:10.1111/myc.12414.
38. Rodrigues, A.M.; de Hoog, G.S.; de Camargo, Z.P. Molecular diagnosis of pathogenic *Sporothrix* species. *PLoS Negl Trop Dis* **2015**, *9*, e0004190, doi:10.1371/journal.pntd.0004190.
39. Mahmoudi, S.; Zaini, F.; Kordbacheh, P.; Safara, M.; Heidari, M. *Sporothrix schenckii* complex in Iran: Molecular identification and antifungal susceptibility. *Med Mycol* **2016**, *54*, 593-599, doi:10.1093/mmy/myw006.
40. de Beer, Z.W.; Wingfield, M.J. Emerging lineages in the Ophiostomatales. In *The Ophiostomatoid Fungi: Expanding Frontiers*, Seifert, K.A., de Beer, Z.W., Wingfield, M.J., Eds.; CBS Biodiversity Series; CBS-KNAW Fungal Biodiversity Centre: Utrecht, The Netherlands, 2013; Volume 12, pp. 21-46.
41. Zhang, Y.; Hagen, F.; Wan, Z.; Liu, Y.; Liu, Y.; Wang, Q.; de Hoog, G.S.; Li, R.; Zhang, J. Two cases of sporotrichosis of the right upper extremity in right-handed patients with diabetes mellitus. *Rev Iberoam Micol* **2016**, *33*, 38-42, doi:10.1016/j.riam.2015.02.001.
42. Nath, R.; Lahon, P.; Timung, L. Molecular identification and phenotypic characterisation of *Sporothrix globosa* from clinical cases of Eastern Assam, North-east India. *Indian J Med Microbiol* **2017**, *35*, 269-273, doi:10.4103/ijmm.IJMM_16_264.
43. Moussa, T.A.; Kadasa, N.M.; Al Zahrani, H.S.; Ahmed, S.A.; Feng, P.; Gerrits van den Ende, A.H.; Zhang, Y.; Kano, R.; Li, F.; Li, S.; et al. Origin and distribution of *Sporothrix globosa* causing sapronoses in Asia. *J Med Microbiol* **2017**, doi:10.1099/jmm.0.000451.
44. Zhao, L.; Cui, Y.; Zhen, Y.; Yao, L.; Shi, Y.; Song, Y.; Chen, R.; Li, S. Genetic variation of *Sporothrix globosa* isolates from diverse geographic and clinical origins in China. *Emerg Microbes Infect* **2017**, *6*, e88, doi:10.1038/emi.2017.75.
45. García Duarte, J.M.; Wattiez Acosta, V.R.; Fornerón Viera, P.M.L.; Aldama Caballero, A.; Gorostiaga Matiauda, G.A.; Rivelli de Oddone, V.B.; Pereira Brunelli, J.G. Esporotricosis trasmitida por gato doméstico. Reporte de un caso familiar. *Revista del Nacional (Itauguá)* **2017**, *9*, 67-76.
46. New, D.; Beukers, A.G.; Kidd, S.E.; Merritt, A.J.; Weeks, K.; van Hal, S.J.; Arthur, I. Identification of multiple species and subpopulations among Australian clinical *Sporothrix* isolates using whole genome sequencing. *Med Mycol* **2018**, doi:10.1093/mmy/myy126.
47. Boechat, J.S.; Oliveira, M.M.E.; Almeida-Paes, R.; Gremiao, I.D.F.; Machado, A.C.S.; Oliveira, R.V.C.; Figueiredo, A.B.F.; Rabello, V.B.S.; Silva, K.B.L.; Zancope-Oliveira, R.M.; et al. Feline sporotrichosis: associations between clinical-epidemiological profiles and phenotypic-genotypic characteristics of the etiological agents in the Rio de Janeiro epizootic area. *Mem Inst Oswaldo Cruz* **2018**, *113*, 185-196, doi:10.1590/0074-02760170407.
48. Córdoba, S.; Isla, G.; Szusz, W.; Vivot, W.; Hevia, A.; Davel, G.; Canteros, C.E. Molecular identification and susceptibility profile of *Sporothrix schenckii sensu lato* isolated in Argentina. *Mycoses* **2018**, *61*, 441-448, doi:10.1111/myc.12760.
49. Macedo-Sales, P.A.; Souto, S.; Destefani, C.A.; Lucena, R.P.; Machado, R.L.D.; Pinto, M.R.; Rodrigues, A.M.; Lopes-Bezerra, L.M.; Rocha, E.M.S.; Baptista, A.R.S. Domestic feline contribution in the transmission

of *Sporothrix* in Rio de Janeiro State, Brazil: a comparison between infected and non-infected populations. *BMC Vet Res* **2018**, *14*, 19, doi:10.1186/s12917-018-1340-4.

50. Fernandes, B.; Caligiorne, R.B.; Coutinho, D.M.; Gomes, R.R.; Rocha-Silva, F.; Machado, A.S.; Santrer, E.F.R.; Assunção, C.B.; Guimarães, C.F.; Laborne, M.S.; et al. A case of disseminated sporotrichosis caused by *Sporothrix brasiliensis*. *Med Mycol Case Rep* **2018**, *21*, 34-36, doi:10.1016/j.mmcr.2018.03.006.
51. Florez-Munoz, S.V.; Alzate, J.F.; Mesa-Arango, A.C. Molecular identification and antifungal susceptibility of clinical isolates of *Sporothrix schenckii* complex in Medellin, Colombia. *Mycopathologia* **2018**, 1-11, doi:10.1007/s11046-018-0310-5.
52. Oliveira, M.M.E.; Almeida-Paes, R.; Corrêa-Moreira, D.; Borba, C.M.; Menezes, R.C.; Freitas, D.F.S.; do Valle, A.C.F.; Schubach, A.O.; Barros, M.B.L.; Nosanchuk, J.D.; et al. A case of sporotrichosis caused by different *Sporothrix brasiliensis* strains: mycological, molecular, and virulence analyses. *Mem Inst Oswaldo Cruz* **2019**, *114*, e190260, doi:10.1590/0074-02760190260.
53. Duangkaew, L.; Yurayart, C.; Limsivilai, O.; Chen, C.; Kasorndorkbua, C. Cutaneous sporotrichosis in a stray cat from Thailand. *Med Mycol Case Rep* **2019**, *23*, 46-49, doi:10.1016/j.mmcr.2018.12.003.
54. Thomson, J.; Trott, D.J.; Malik, R.; Galgut, B.; McAllister, M.M.; Nimmo, J.; Renton, D.; Kidd, S.E. An atypical cause of sporotrichosis in a cat. *Med Mycol Case Rep* **2019**, *23*, 72-76, https://doi.org/10.1016/j.mmcr.2019.01.004.
55. Zhang, M.; Li, F. Fast diagnosis of sporotrichosis caused by *Sporothrix globosa*, *Sporothrix schenckii*, and *Sporothrix brasiliensis* based on multiplex real-time PCR. *PLoS Negl Trop Dis* **2019**, *13*, e0007219, doi:10.1371/journal.pntd.0007219.
56. Gong, J.; Zhang, M.; Wang, Y.; Li, R.; He, L.; Wan, Z.; Li, F.; Zhang, J. Population structure and genetic diversity of *Sporothrix globosa* in China according to 10 novel microsatellite loci. *J Med Microbiol* **2019**, *68*, 248-254, doi:10.1099/jmm.0.000896.
57. Etchecopaz, A.N.; Lanza, N.; Toscanini, M.A.; Devoto, T.B.; Pola, S.J.; Daneri, G.L.; Iovannitti, C.A.; Cuestas, M.L. Sporotrichosis caused by *Sporothrix brasiliensis* in Argentina: Case report, molecular identification and in vitro susceptibility pattern to antifungal drugs. *J Mycol Med* **2019**, 100908, doi:10.1016/j.mycmed.2019.100908.
58. Li, J.; Zhan, P.; Jiang, Q.; Gao, Y.; Jin, Y.; Zhang, L.; Luo, Y.; Fan, X.; Sun, J.; de Hoog, S. Prevalence and antifungal susceptibility of *Sporothrix* species in Jiangxi, central China. *Med Mycol* **2019**, *57*, 954-961, doi:10.1093/mmy/myy163.
59. Kamal Azam, N.K.; Selvarajah, G.T.; Santhanam, J.; Abdul Razak, M.F.; Ginsapu, S.J.; James, J.E.; Suetrong, S. Molecular epidemiology of *Sporothrix schenkii* isolates in Malaysia. *Med Mycol* **2020**, *58*, 617-625, doi:10.1093/mmy/myz106.
60. Rasamoelina, T.; Maubon, D.; Raharolahy, O.; Razanakoto, H.; Rakotozandrindrainy, N.; Rakotomalala, F.A.; Bailly, S.; Sendrasoa, F.; Ranaivo, I.; Andrianarison, M.; et al. Sporotrichosis in the Highlands of Madagascar, 2013-2017(1). *Emerg Infect Dis* **2019**, *25*, 1893-1902, doi:10.3201/eid2510.190700.
61. Makri, N.; Paterson, G.K.; Gregge, F.; Urquhart, C.; Nuttall, T. First case report of cutaneous sporotrichosis (*Sporothrix* species) in a cat in the UK. *JFMS Open Rep* **2020**, *6*, 2055116920906001, doi:10.1177/2055116920906001.
62. do Monte Alves, M.; Pipolo Milan, E.; da Silva-Rocha, W.P.; Soares de Sena da Costa, A.; Araújo Maciel, B. Fatal pulmonary sporotrichosis caused by *Sporothrix brasiliensis* in Northeast Brazil. **2020**, *14*, e0008141, doi:10.1371/journal.pntd.0008141.
63. Valeriano, C.A.T.; Lima-Neto, R.G.; Inácio, C.P.; Rabello, V.B.S.; Oliveira, E.P.; Zancopé-Oliveira, R.M.; Almeida-Paes, R.; Neves, R.P.; de Oliveira, M.M.E. Is *Sporothrix chilensis* circulating outside Chile? *PLoS Negl Trop Dis* **2020**, *14*, e0008151, doi:10.1371/journal.pntd.0008151.
64. Monno, R.; Brindicci, G.; Romeo, O.; De Carolis, E.; Criseo, G.; Sanguinetti, M.; Fumarola, L.; Ingravallo, G.; Mariani, M.; Monno, L. Infection caused by *Sporothrix schenckii*: an autochthonous case in Bari, Southern Italy. *Eur J Clin Microbiol Infect Dis* **2020**, *39*, 2457-2460, doi:10.1007/s10096-020-03939-z.
65. de Carvalho, J.A.; Hagen, F.; Fisher, M.C.; de Camargo, Z.P.; Rodrigues, A.M. Genome-wide mapping

using new AFLP markers to explore intraspecific variation among pathogenic *Sporothrix* species. *PLoS Negl Trop Dis* **2020**, *14*, e0008330, doi:10.1371/journal.pntd.0008330.

66. Gonsales, F.F.; Fernandes, N.C.C.A.; Mansho, W.; Montenegro, H.; Benites, N.R. Direct PCR of lesions suggestive of sporotrichosis in felines. *Arq Bras Med Vet Zootec [online]* **2020**, *72*, 2002-2006.

67. Zhang, M.; Li, F.; Gong, J.; Yang, X.; Zhang, J.; Zhao, F. Development and evaluation of a real-time polymerase chain reaction for fast diagnosis of sporotrichosis caused by *Sporothrix globosa*. *Med Mycol* **2020**, *58*, 61-65, doi:10.1093/mmy/myz029.

68. Rudramurthy, S.M.; Shankarnarayan, S.A.; Hemashetter, B.M.; Verma, S.; Chauhan, S.; Nath, R.; Savio, J.; Capoor, M.; Kaur, H.; Ghosh, A.K.; et al. Phenotypic and molecular characterisation of *Sporothrix globosa* of diverse origin from India. *Braz J Microbiol* **2021**, *52*, 91-100, doi:10.1007/s42770-020-00346-6.

69. da Cruz Bahiense Rocha, I.; Terra, P.P.D.; Cardoso de Oliveira, R.; Lubianca Zanotti, R.; Falqueto, A.; de Camargo, Z.P.; Rodrigues, A.M. Molecular-based assessment of diversity and population structure of *Sporothrix* spp. clinical isolates from Espírito Santo-Brazil. **2021**, *64*, 420-427, doi:10.1111/myc.13230.

70. Ramírez-Soto, M.C.; Aguilar-Ancori, E.G.; Quispe-Ricalde, M.A.; Muñiz-Duran, J.G.; Quispe-Florez, M.M.; Chinen, A. Molecular identification of *Sporothrix* species in a hyperendemic area in Peru. *J Infect Public Health* **2021**, *14*, 670-673, doi:10.1016/j.jiph.2021.02.005.

71. Maschio-Lima, T.; Marques, M.D.R.; Lemes, T.H.; Brizzotti-Mazuchi, N.S.; Caetano, M.H.; de Almeida, B.G.; Bianco, L.M.; Monteiro, R.C.; Rodrigues, A.M.; de Camargo, Z.P.; et al. Clinical and epidemiological aspects of feline sporotrichosis caused by *Sporothrix brasiliensis* and *in vitro* antifungal susceptibility. *Vet Res Commun* **2021**, doi:10.1007/s11259-021-09795-2.

72. de Carvalho, J.A.; Beale, M.A.; Hagen, F.; Fisher, M.C.; Kano, R.; Bonifaz, A.; Toriello, C.; Negroni, R.; Rego, R.S.M.; Gremiao, I.D.F.; et al. Trends in the molecular epidemiology and population genetics of emerging *Sporothrix* species. *Stud Mycol* **2021**, *100*, 100129, doi:10.1016/j.simyco.2021.100129.
